# Superior-head automated external defibrillator placement during single-rescuer cardiopulmonary resuscitation: a randomized crossover simulation study and retrospective multicenter cohort

**DOI:** 10.3389/fpubh.2026.1927906

**Published:** 2026-09-03

**Authors:** Daojian Xu, Jingchen Zhang, Honghang Lin, Qiqi Cai, Sheng Zhang, Jueyue Yan

**Affiliations:** 1 Department of Emergency Medicine, Taizhou First People’s Hospital, School of Medicine, Taizhou University Affiliated Huangyan Hospital, Wenzhou Medical University Huangyan Hospital, Taizhou, Zhejiang, China; 2 Intensive Care Unit, The First Affiliated Hospital, College of Medicine, Zhejiang University, Hangzhou, Zhejiang, China

**Keywords:** automated external defibrillator, cardiopulmonary resuscitation, chest compression fraction, defibrillation, human factors, out-of-hospital cardiac arrest, simulation training, time to shock

## Abstract

**Background:**

During single-rescuer cardiopulmonary resuscitation (CPR), one rescuer must sustain chest compressions while operating an automated external defibrillator (AED), yet the optimal physical position of the AED within the rescuer’s working area is uncertain. The study evaluated whether superior-head AED placement improves the single-rescuer defibrillation workflow.

**Methods:**

The study combined a randomized four-sequence crossover manikin simulation (32 healthcare providers; 128 evaluable trials) comparing four prespecified AED positions—superior-head placement (position D) versus alternative placements (positions A–C)—with a retrospective cohort of 213 AED-treated adult cardiac-arrest cases from six centers (57 with position D, 156 with other positions). The primary simulation outcome was compression-to-first-shock time; the primary clinical outcome was the collapse-to-first-shock interval. Secondary outcomes included AED-related hands-off time, chest compression fraction, NASA Task Load Index workload, and procedural safety.

**Results:**

In the simulation, position D shortened compression-to-first-shock time by 14.8–26.3 s relative to the other positions (all Holm-adjusted *p* < 0.001), reduced AED-related hands-off time, increased chest compression fraction (83% vs. 78–80%), and lowered perceived workload (all *p* < 0.001), without increasing critical errors; over-body actions were least frequent with position D. In the retrospective cohort, the adjusted median difference in the collapse-to-first-shock interval was −0.42 min (approximately 25 s; 95% CI, −0.91 to 0.07; *p* = 0.092); estimates consistently favored position D across sensitivity, restricted-cohort, weighted, and leave-one-center-out analyses, although every confidence interval crossed the null. Patient outcomes did not differ by AED position.

**Conclusion:**

Superior-head AED placement improved the efficiency, compression continuity, and workload of simulated single-rescuer CPR. The retrospective clinical findings were directionally consistent but statistically inconclusive and should be regarded as hypothesis-generating rather than confirmatory. Prospective validation is required before clinical implementation.

## Introduction

Out-of-hospital cardiac arrest (OHCA) remains a major global health challenge, with pooled survival to hospital discharge remaining below 10% despite improvements in resuscitation systems. Early recognition, immediate high-quality cardiopulmonary resuscitation (CPR), and prompt use of an automated external defibrillator (AED) are therefore central components of the chain of survival (1–3).

For patients with a shockable rhythm, the probability of survival is strongly time dependent. Rapid defibrillation by trained first responders or public-access AED programs can substantially improve survival (4, 5). During AED deployment, however, maintaining coronary and cerebral perfusion also requires chest-compression interruptions to be kept as short as possible. Higher chest compression fraction has been associated with improved survival in ventricular fibrillation, whereas prolonged preshock and perishock pauses have been associated with lower survival (6, 7). Contemporary resuscitation guidelines consequently emphasize early defibrillation together with continuous, high-quality compressions and minimization of avoidable pauses (2).

This coordination is particularly demanding during single-rescuer CPR. One rescuer must continue compressions while activating the AED, exposing the chest, applying the electrode pads, managing the cable, following rhythm-analysis prompts, clearing the patient, and delivering the shock. AED rhythm analysis and shock preparation account for a measurable proportion of compression interruption time in clinical resuscitation (8). Previous research has largely addressed AED accessibility, device usability, prompting, training, and electrode-pad placement; device design alone can produce clinically relevant differences in time to shock (9). By contrast, little evidence is available on whether the physical position of the AED within the rescuer’s immediate working area can improve access, reduce task interference, or shorten the pathway to shock without compromising procedural safety.

To address this practical knowledge gap, the study combined a randomized crossover simulation study with a retrospective multicenter clinical cohort study. The simulation compared four prespecified AED positions during single-rescuer CPR and evaluated compression-to-first-shock time, AED-related hands-off time, chest compression fraction, perceived workload, and procedural safety. The clinical study then examined whether superior-head AED placement (position D) was associated with shorter collapse-to-first-shock and AED-proximal process intervals in routine resuscitation. This study hypothesized that position D would streamline the single-rescuer workflow, shorten time to shock and AED-related interruptions, preserve chest compressions, and reduce workload without increasing critical errors or unsafe over-body actions.

## Methods

### Study design

Two complementary studies were conducted to evaluate AED placement during single-rescuer CPR. First, a randomized four-sequence crossover simulation compared four prespecified AED positions within the same participants. Second, a retrospective multicenter cohort study examined whether superior-head position D was associated with shorter clinical process intervals than other AED positions. The simulation was designed to estimate position-specific procedural effects under controlled conditions, whereas the clinical study evaluated associations in routine resuscitation care.

### Sample-size determination

No formal *a priori* sample-size or power calculation was performed for either study component. The simulation sample was pragmatic and based on the number of eligible healthcare providers who consented and could complete all four crossover periods during January 2026. The 32 participants contributed 32 paired observations for each AED position, yielding 128 evaluable trials. The retrospective cohort included all identifiable records meeting the prespecified eligibility criteria from the six participating centers between January 2018 and December 2025, resulting in 213 patients. The clinical cohort was therefore availability-based and intended primarily to estimate the direction and magnitude of associations.

The crossover component was prepared with reference to the CONSORT extension for randomized crossover trials, and the retrospective cohort component was prepared in accordance with the STROBE reporting recommendations (10, 11). The retrospective cohort was conducted at six participating centers in China: the First Affiliated Hospital, Zhejiang University School of Medicine; Quzhou Kecheng People’s Hospital; the People’s Hospital of Pingyang; Taizhou First People’s Hospital; Sanmen People’s Hospital; and the People’s Hospital of Cangnan. The retrospective cohort protocol was approved by the institutional ethics committees of the First Affiliated Hospital, Zhejiang University School of Medicine (IIT20230253B-R2 and No. 2021-IIT-1163), Quzhou Kecheng People’s Hospital (KCYY2025-K53), the People’s Hospital of Pingyang (IRB-2025-025), Taizhou First People’s Hospital (2025-KY133-01), Sanmen People’s Hospital (2512261025650), and the People’s Hospital of Cangnan (20250588). The requirement for individual informed consent was waived by the relevant ethics committees for the retrospective cohort because it involved the secondary use of existing clinical data and source materials. By contrast, all healthcare-provider participants provided written informed consent before enrolment in the prospective manikin-based crossover simulation. The simulation study was not prospectively registered because it did not evaluate a clinical intervention or patient health outcome. All study procedures were conducted in accordance with the Declaration of Helsinki.

### Randomized crossover simulation study

Participants were recruited in January 2026 from physicians, nurses, and emergency medical technicians with experience relevant to basic life support or AED use. Eligible individuals were required to be able and willing to complete all four single-rescuer AED-positioning simulation periods. Of the 36 individuals assessed for eligibility, four were not enrolled: one declined participation, one had a recent musculoskeletal condition that could interfere with safe performance of the simulation tasks, one had participated in pilot testing, and one was unable to commit to completion of all four study periods. The remaining 32 participants were enrolled and randomly assigned in equal numbers to one of four prespecified crossover sequences.

Participants were randomly assigned in a 1:1:1:1 ratio to one of four prespecified counterbalanced sequences: A–B–D–C, B–C–A–D, C–D–B–A, or D–A–C–B. Each sequence included eight participants. Every participant completed each of the four AED-positioning scenarios once over four study periods, yielding 128 evaluable trials. Sequence assignment was implemented by the study team according to a balanced schedule established before enrolment. The available records do not document the random-number generation method or software, the identity of the individual allocator, or an independent allocation-concealment mechanism; therefore, formal allocation concealment cannot be independently verified. Before each period, the manikin, AED, electrode pads, and surrounding simulation environment were reset under standardized conditions. No formal washout procedure was prespecified because the intervention consisted solely of changing the physical position of the AED; participants were allowed sufficient time to recover and prepare before beginning the next scenario. Blinding of participants and simulation personnel was not feasible because the assigned AED position was visually apparent. Outcome assessment was therefore based on prespecified objective time definitions and standardized procedural criteria to minimize assessment bias. Participants did not receive performance feedback between study periods.

**Figure 1:**
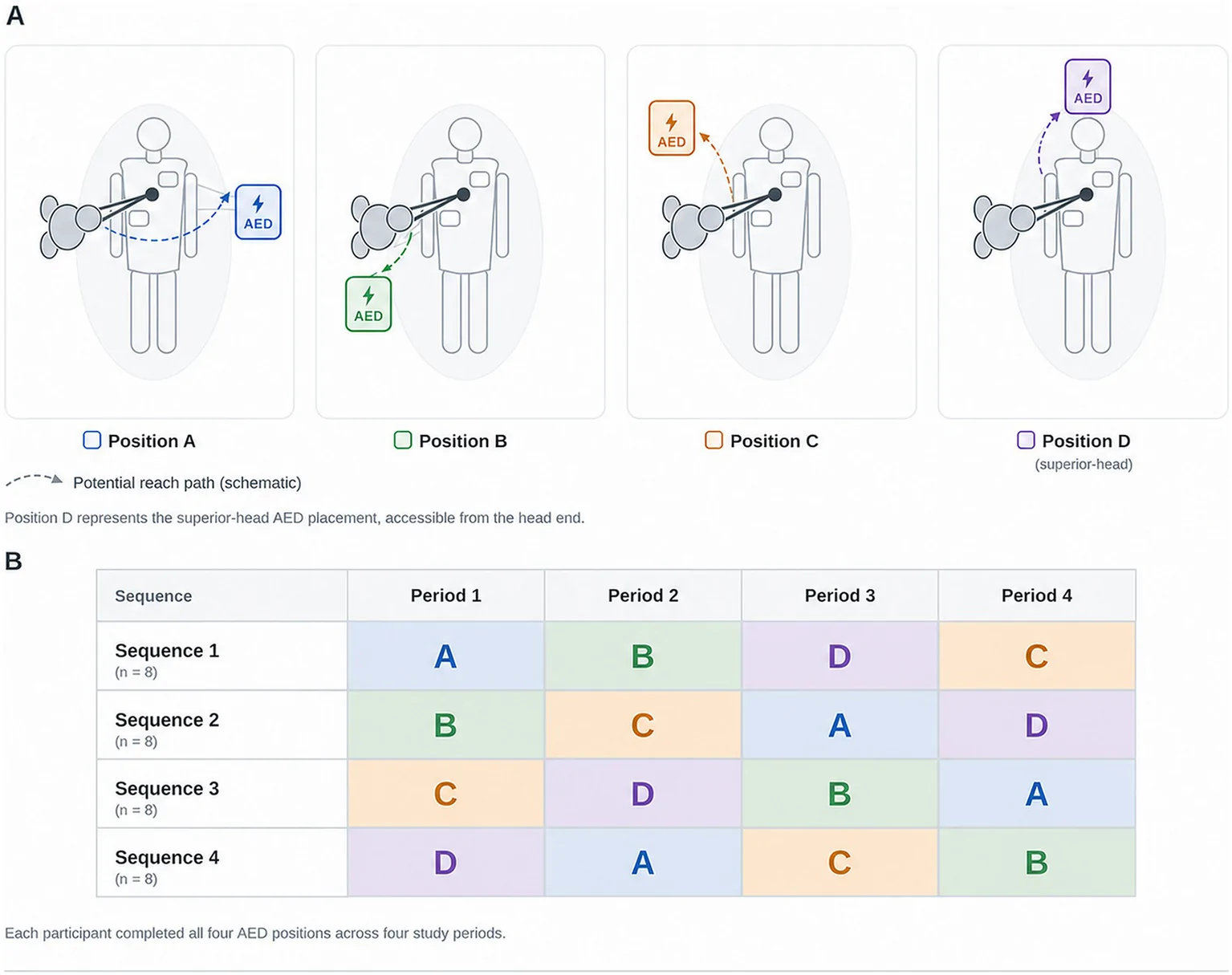
Study design of the randomized four-sequence crossover AED simulation. **(A)** The four prespecified AED positions evaluated during single-rescuer CPR. Position D denotes superior-head placement above the patient’s head, accessible from the head end; positions A–C represent the alternative placements. Color-matched arrows illustrate the potential reach path from the rescuer’s working position toward each AED location. These paths are schematic and are intended to illustrate differences in operator–device geometry; they do not represent measured movement trajectories, distances, or frequencies. **(B)** Counterbalanced four-sequence crossover design. Participants were randomly assigned 1:1:1:1 to sequence A–B–D–C, B–C–A–D, C–D–B–A, or D–A–C–B (*n* = 8 each) and completed each AED position once across four study periods. AED, automated external defibrillator; CPR, cardiopulmonary resuscitation.

The four AED locations were prespecified before enrolment (Figure 1). Position D placed the AED above the patient’s head, whereas positions A–C represented the alternative placements shown in Figure 1A. All scenarios used the same JW3201 full-body adult CPR manikin (Beijing Medical Model, Beijing, China) and a BeneHeart D1 Pro AED platform (Shenzhen Mindray Bio-Medical Electronics Co., Ltd., Shenzhen, China) under standardized simulation conditions with a simulated shockable rhythm. The BeneHeart D1 Pro was used with its corresponding preconnected adult multifunction defibrillation pads; the manufacturer reports a pad-cable length of approximately 2.1 m for this system. In all simulation trials, the pads were applied in the standard anterolateral configuration. This configuration permitted pad application with the AED at position D without repositioning the device. Anteroposterior pad placement was not evaluated. The AED, electrode-pad packaging and configuration, cable arrangement, and device settings were kept constant across all four positioning scenarios. At the start of each period, the participant was positioned beside the manikin and the AED was placed in the assigned location. After a standardized start command, the participant performed single-rescuer CPR and followed the AED prompts until delivery of the first simulated shock. Participants were instructed to follow standard basic life support procedures and minimize interruptions in chest compressions. The equipment and study environment were reset between periods. All participants received the same brief equipment orientation, without position-specific coaching or performance feedback. Compression-to-first-shock time was defined as the interval from the first chest compression to the first simulated shock. Time points and procedural events were determined from video recordings and available AED event records using a prespecified review protocol.

### Simulation outcomes

The primary simulation outcome was compression-to-first-shock time, defined as the interval from the first chest compression to delivery of the first shock. Key secondary outcomes were AED-related hands-off time, chest compression fraction, and perceived workload. AED-related hands-off time comprised the interruption attributable to AED activation, pad handling and placement, cable management, rhythm analysis, clearing, and shock delivery during the compression-to-first-shock observation window. Chest compression fraction was calculated as chest-compression time divided by the compression-to-first-shock interval.

Perceived workload was assessed immediately after each AED-position scenario, before the next study period, using the Chinese-language raw National Aeronautics and Space Administration Task Load Index (NASA-TLX) (12). The raw score was calculated as the unweighted mean of the mental demand, physical demand, temporal demand, performance, effort, and frustration domains, each scored from 0 to 100. For the performance domain, higher scores indicated poorer perceived performance; therefore, higher composite scores indicated greater perceived workload.

Prespecified procedural-safety outcomes were at least one critical error and an over-body action during the trial. A critical error was defined as an action or omission that could materially delay or compromise AED operation, rhythm analysis, shock delivery, or procedural safety. Prespecified critical errors included: (1) failure to activate the AED or inadvertent device shutdown; (2) failure to attach both electrode pads or connect the pad cable correctly; (3) pad placement requiring repositioning before rhythm analysis or shock delivery; (4) cable entanglement or disconnection that prevented or interrupted AED operation; (5) continued contact with the patient during rhythm analysis; (6) failure to clear the patient before shock delivery; and (7) failure to deliver a shock when advised by the AED. An over-body action or a temporary interruption in chest compressions was not classified as a critical error unless it met one of these criteria. An over-body action was defined separately as reaching or moving across the patient’s torso to operate or reposition the AED, electrode pads, or cables. Each trial was classified according to whether at least one critical error occurred. Video recordings were independently reviewed by two trained clinical investigators with resuscitation experience using these prespecified criteria. Blinding to AED position was not feasible because the assigned position was visible. Disagreements were resolved by consensus.

### Statistical analysis of the simulation study

Continuous variables were summarized as mean (SD) or median (IQR), as appropriate, and categorical variables as number (percentage). Participant characteristics across randomization sequences were compared descriptively using one-way analysis of variance for approximately normally distributed variables, the Kruskal–Wallis test for skewed variables, and the Fisher–Freeman–Halton exact test for multicategory categorical variables. Baseline *p* values were not used to judge randomization success or select model covariates.

Unadjusted repeated distributions across the four AED positions were compared using the Friedman test. The prespecified primary analysis of compression-to-first-shock time used a linear mixed-effects model fitted by maximum likelihood. AED position, study period, and randomization sequence were entered as fixed effects, and participant was included as a random intercept. Secondary continuous outcomes were analzsed using the same fixed- and random-effects structure and restricted maximum-likelihood estimation. Overall position, period, and sequence effects were assessed using joint Wald chi-square tests.

Three prespecified contrasts compared position D separately with positions A, B, and C. Within each outcome, the three contrast *p* values were adjusted using the Holm step-down procedure to control the family-wise error rate (13). Effect estimates are reported as position D minus the comparator. Thus, negative differences favor position D for time-based outcomes and workload, whereas positive differences favor position D for chest compression fraction. Reported 95% Wald confidence intervals were nominal and were not multiplicity adjusted. Repeated-measures analysis of variance with Greenhouse–Geisser correction was used as a supportive omnibus analysis, and partial eta-squared quantified the magnitude of position effects.

For paired binary safety outcomes, overall differences among positions were assessed using Cochran’s *Q* test. Each comparator was then assessed against position D using a two-sided exact McNemar test. Matched odds ratios were calculated from discordant pairs, and the three comparison *p* values were Holm adjusted separately within each safety endpoint; exact 95% confidence intervals were not multiplicity adjusted.

Exploratory order analyses evaluated position-by-period and position-by-sequence interactions. To avoid rank deficiency in the balanced four-sequence design, position-by-period models omitted sequence and position-by-sequence models omitted period. Linear period terms were used to assess learning trends, the period 4-versus-period 3 contrast assessed late-period fatigue, and the within-participant position D advantage was compared across sequences. No separate first-order carryover coefficient was estimated because the four sequences collectively included each of the 12 ordered first-order transitions once.

### Retrospective multicenter cohort study

Adult cardiac-arrest records were screened retrospectively from six participating centers between January 2018 and December 2025. Potential cases were identified from available institutional cardiac-arrest records, emergency medical service documentation, AED event logs, and linked clinical records. Records were eligible when an AED-treated arrest had an identifiable rhythm trajectory and a clearly documented single-rescuer AED-assisted phase before the first rhythm analysis. Cases were excluded if the first shock was delivered using a manual defibrillator or if the AED was applied only after arrival of the full professional team. Patients with an initially non-shockable rhythm were eligible if a shockable rhythm subsequently developed during the identifiable phase and both the first-shock time and AED position could be verified. Records were excluded when AED or rescuer position, or the first-shock time, could not be classified reliably because of missing, conflicting, or insufficient documentation. Records were linked using available patient identifiers and event dates, and duplicate entries were removed during screening. Screening followed a prespecified review form; uncertain cases were resolved by consensus after review of the complete available record.

### Exposure classification and clinical variables

The exposure was superior-head AED placement (position D) versus any other placement (positions A–C). Position D was defined as placement above the patient’s head and accessible from the head end. Two trained clinical investigators independently classified AED position using all available contemporaneous documentation, including video or photographic evidence, AED event records, emergency department or EMS documentation, dispatch records, and clinical records. Each reviewer recorded a provisional classification before comparison or consensus review. Direct visual evidence was prioritized; otherwise, classification required concordant information across available records. Classifications supported by direct visual evidence or concordant contemporaneous documentation were designated as high confidence. Cases with undocumented or conflicting positions, or repositioning before the first shock, were excluded. The 17 discrepant classifications were resolved by consensus after joint review of the complete available record. Blinding to AED position was not feasible because the assigned position was evident from the source documentation.

Prespecified covariates for the primary clinical model were age, sex, witnessed-arrest status, bystander CPR, arrest location, first-rescuer type, participating center, and calendar period. Additional descriptive variables included comorbidities, arrest etiology, initial rhythm, witness type, AED source and mode, collapse-to-CPR interval, collapse-to-AED-arrival interval, and emergency medical services response interval. Patient outcomes were return of spontaneous circulation, 24-h survival, 7-day survival, survival to hospital discharge, and favorable neurological outcome at discharge, defined as Cerebral Performance Category 1 or 2.

### Ascertainment of retrospective clinical time-to-shock intervals

Clinical time to shock (TTS) was operationalized separately from the simulation compression-to-first-shock time. The primary retrospective TTS measure was the collapse-to-first-shock interval, calculated as the timestamp of the first AED-delivered shock minus the estimated time of collapse. The prespecified binary outcome indicated whether this interval was 8 min or less, reflecting the historical 8-min defibrillation-response benchmark used in emergency medical systems, while recognizing that shorter intervals are associated with greater survival (14). The collapse-to-first-shock interval was prespecified as the primary clinical process outcome because it was the most consistently verifiable and clinically interpretable interval across the six participating centers and was available for all 213 included patients. It captured the entire pre-shock response pathway, including arrest recognition, emergency activation, AED retrieval, rescuer arrival, device deployment, and shock delivery. Because AED position could influence only the portion after the AED reached the patient, this interval was interpreted as a system-level process outcome rather than a position-specific mechanistic measure. It was not treated as interchangeable with the simulation interval, which began at the first chest compression.

The collapse timestamp was abstracted from the most contemporaneous available source, including video, emergency-call or dispatch records, witness documentation, and EMS records. When multiple sources were available, directly time-stamped records were prioritized and discrepancies were resolved by review of the complete event record. The first-shock timestamp was obtained preferentially from the time-stamped AED event file; synchronized video or EMS documentation was used only when the AED record was unavailable but the time could still be verified reliably. All timestamps were reconciled to the local event date and time before analysis. The collapse-to-first-shock interval was calculated in seconds from the adjudicated timestamps and converted to minutes for reporting.

A TTS value was considered verifiable only when both component timestamps referred to the same arrest episode and occurred in a chronologically plausible sequence. Records were excluded if the first-shock time could not be confirmed, if available time records were materially inconsistent, or if the AED event record was insufficient to establish shock timing. When sources disagreed, all available records were reviewed using the prespecified source hierarchy, with priority given to directly time-stamped AED or video records; uncertain cases were resolved by consensus. Reviewers could not be blinded to AED position when it was evident from the source documentation. Because formal inter-rater agreement was not prespecified, no agreement statistic was reported.

For patients with complete AED records, two position-proximal intervals were derived. The AED-arrival-to-first-shock interval was measured from the documented arrival of the AED at the patient to delivery of the first shock, whereas the AED-activation-to-pad-placement interval was measured from device activation to completion of pad placement. AED arrival was determined from the most reliable contemporaneous source, prioritizing video when available. Activation and shock times were obtained from AED event logs, and pad-placement completion was identified from available event markers or corroborating video review. These intervals were analyzed only when all required timestamps were available and chronologically consistent. These position-proximal intervals were designated as secondary outcomes because complete and chronologically consistent AED-log data were available for only 176 patients (48 with position D and 128 with other positions). In addition, pad-placement completion was not uniformly recorded across AED models and sometimes required corroborating video review. Selecting either interval as the primary outcome would therefore have restricted the analytic cohort and potentially increased selection bias. These measures were consequently used as supportive mechanistic outcomes.

### Statistical analysis of the clinical cohort

Continuous variables were summarized as mean (SD) or median (IQR), and categorical variables as number (percentage) using the available non-missing denominator. Age was compared using Welch’s *t* test, skewed time intervals using the Mann–Whitney *U* test, and categorical variables using Pearson’s chi-square test when expected counts were adequate. Arrest location was compared using the Fisher–Freeman–Halton exact test. Standardized mean differences were calculated for baseline variables; category-specific values were reported for multicategory variables, and skewed intervals were evaluated on the log scale. An absolute standardized mean difference of 0.20 or greater was considered a moderate imbalance for descriptive purposes.

The primary continuous analysis estimated the conditional median difference in collapse-to-first-shock interval between position D and other positions using median regression at the 50th percentile (15). The primary model included age, sex, witnessed arrest, bystander CPR, arrest location, first-rescuer type, center, and calendar period as fixed covariates. Confidence intervals were obtained from 5,000 patient-level nonparametric bootstrap resamples. Negative coefficients indicated shorter intervals with position D. A secondary model additionally adjusted for the collapse-to-AED-arrival interval among patients with this variable available.

Achievement of a collapse-to-first-shock interval of 8 min or less was analyzed using logistic regression with the same adjustment sets; odds ratios greater than 1 favored position D. In the complete AED-log cohort, the AED-arrival-to-first-shock interval was analyzed using median regression with the primary covariate set. Unadjusted continuous comparisons used the Wilcoxon–Mann–Whitney test, with Hodges–Lehmann location-shift estimates reported as effect estimates (16).

Sensitivity analyses repeated the primary models in a high-confidence position cohort, a cohort with only one rescuer before the first shock, a witnessed-arrest cohort, an initial-shockable-rhythm cohort, and a strict cohort meeting all four criteria. Covariates with no within-cohort variation were omitted. The primary model was also repeated after omitting each center in turn. An inverse-probability-weighted analysis estimated the weighted mean difference in collapse-to-first-shock interval; stabilized weights were truncated at the 1st and 99th percentiles, and balance assessment followed established recommendations for propensity-score weighting (17). An absolute standardized mean difference <0.10 after weighting was considered indicative of adequate balance. The IPW analysis estimated the weighted mean difference in collapse-to-first-shock interval and was interpreted as supportive rather than confirmatory.

Exploratory patient outcomes were first summarized using unadjusted odds ratios with log-Wald confidence intervals. Adjusted associations were estimated using parsimonious Firth penalized logistic-regression models to reduce small-sample bias and address sparse-event separation (18). Models included age, witnessed-arrest status, and initial shockable rhythm. Profile penalized-likelihood confidence intervals and two-sided *p* values were reported. Center was not included because of sparse outcome events. These analyses were exploratory and were not adjusted for multiple comparisons.

All statistical tests were two-sided, with a nominal significance level of 0.05. Analyses requiring specific interval data were restricted to participants with complete information for the relevant variables. Missing values were not imputed. All analyses were performed using R statistical software (version 4.6.0; R Foundation for Statistical Computing, Vienna, Austria).

## Results

### Study populations and cohort characteristics

**Figure 2:**
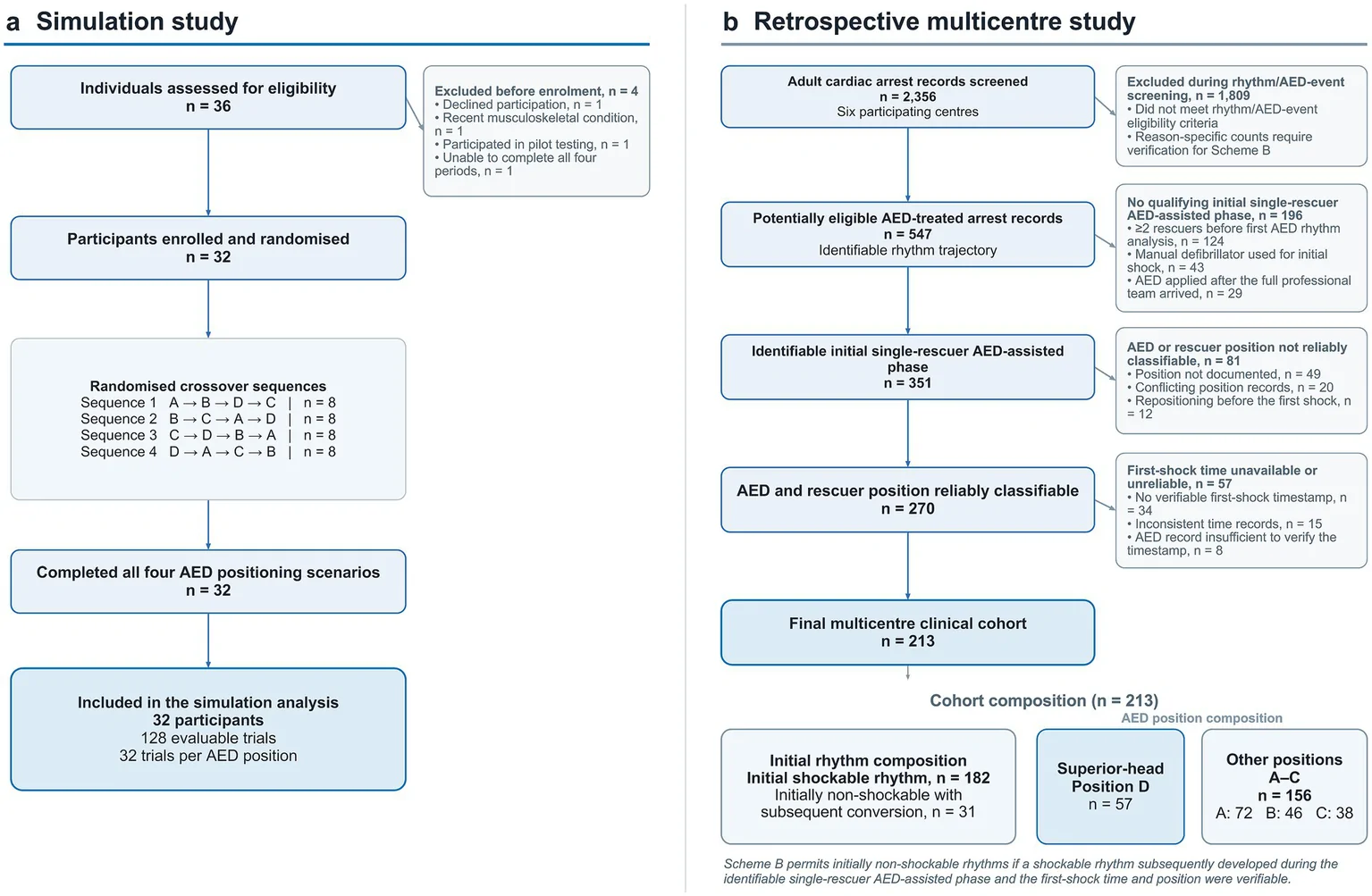
Participant and case flow through the two-part study. **(a)** Simulation study. Of 36 individuals assessed for eligibility, 4 were excluded before enrolment (declined participation, recent musculoskeletal condition, prior pilot participation, and inability to complete all four periods; *n* = 1 each). The 32 enrolled participants were randomized in equal numbers to one of four counterbalanced crossover sequences, completed all four AED-positioning scenarios, and contributed 128 evaluable trials (32 per AED position). **(b)** Retrospective multicenter study. Of 2,356 adult cardiac-arrest records screened across six centers, 1,809 were excluded during rhythm/AED-event screening, leaving 547 potentially eligible AED-treated records; 196 lacked a qualifying initial single-rescuer AED-assisted phase, 81 had an AED or rescuer position that could not be reliably classified, and 57 had an unavailable or unreliable first-shock time. The final cohort comprised 213 patients (182 with an initial shockable rhythm; 31 with subsequent conversion), including 57 treated with superior-head position D and 156 with positions A–C (A, 72; B, 46; C, 38). AED, automated external defibrillator.

Of 36 individuals assessed for the simulation study, four were excluded and 32 were enrolled and randomized, with eight assigned to each of four counterbalanced sequences. All 32 participants completed each of the four AED-position scenarios, yielding 128 evaluable trials (Figures 1A,B, 2a). The participants had a mean age of 31.1 years (SD, 3.3), 18 (56.3%) were women, 29 (90.6%) had previously received basic life support/AED training, and 23 (71.9%) had previous real-life CPR experience (Table 1). Sequence-specific characteristics are reported in Supplementary Table S1.

**Table 1:** Participant characteristics by randomization sequence.

Characteristic	Overall (*n* = 32)	Sequence 1 (*n* = 8)	Sequence 2 (*n* = 8)	Sequence 3 (*n* = 8)	Sequence 4 (*n* = 8)	Group-comparison *p* value
Age, years	31.1 ± 3.3	28.8 ± 2.9	31.6 ± 3.0	32.2 ± 3.1	31.8 ± 3.4	0.125
Female sex	18 (56.3)	6 (75.0)	3 (37.5)	5 (62.5)	4 (50.0)	0.640
Height, cm	169.0 ± 6.9	166.4 ± 7.5	170.6 ± 7.0	169.2 ± 7.7	169.9 ± 5.9	0.652
Body mass index, kg/m^2^	22.6 ± 1.6	21.5 ± 1.6	23.4 ± 1.1	22.5 ± 1.7	23.1 ± 1.5	0.086
Profession						0.538
Physician	11 (34.4)	4 (50.0)	1 (12.5)	3 (37.5)	3 (37.5)	
Nurse	17 (53.1)	3 (37.5)	6 (75.0)	3 (37.5)	5 (62.5)	
Emergency medical technician	4 (12.5)	1 (12.5)	1 (12.5)	2 (25.0)	0 (0.0)	
Clinical experience, years	7.0 [5.0–9.0]	4.5 [2.8–6.2]	7.5 [5.8–9.5]	8.5 [5.8–10.2]	7.5 [5.8–9.2]	0.115
Previous BLS/AED training	29 (90.6)	8 (100.0)	6 (75.0)	8 (100.0)	7 (87.5)	0.587
Time since last BLS/AED training, months*	10.0 [7.0–16.0]	9.0 [5.8–13.5]	11.5 [7.5–18.5]	11.0 [7.5–16.5]	11.0 [8.0–14.0]	0.903
Previous real-life CPR experience	23 (71.9)	7 (87.5)	4 (50.0)	7 (87.5)	5 (62.5)	0.336
Previous real-life AED use	10 (31.3)	4 (50.0)	1 (12.5)	3 (37.5)	2 (25.0)	0.589
Right-hand dominant	30 (93.8)	8 (100.0)	6 (75.0)	8 (100.0)	8 (100.0)	0.226

Data are mean ± SD, median [IQR], or *n* (%). *Calculated among the 29 participants with previous BLS/AED training. Approximately normally distributed continuous variables were compared using one-way ANOVA; skewed variables were compared using the Kruskal–Wallis test; and categorical variables were compared using the Fisher–Freeman–Halton exact test. Baseline *p* values are descriptive, were not adjusted for multiplicity, and were not used to assess the success of randomization or to select covariates.

Sequence orders: Sequence 1, A–B–D–C; Sequence 2, B–C–A–D; Sequence 3, C–D–B–A; Sequence 4, D–A–C–B.

AED, automated external defibrillator; BLS, basic life support; CPR, cardiopulmonary resuscitation; EMT, emergency medical technician; IQR, interquartile range; SD, standard deviation.

Among 2,356 adult cardiac-arrest records screened across six centers, 1,809 were excluded during rhythm/AED-event screening, leaving 547 potentially eligible records with an identifiable rhythm trajectory. A further 196 records lacked a qualifying initial single-rescuer AED-assisted phase, 81 had an AED or rescuer position that could not be reliably classified, and 57 had an unavailable or unreliable first-shock time. Thus, among the 351 otherwise qualifying cases with an identifiable initial single-rescuer AED-assisted phase, 138 (39.3%) were excluded because AED position (*n* = 81) or first-shock time (*n* = 57) could not be reliably verified. The final clinical cohort therefore comprised 213 patients (Figure 2b), including 57 treated with superior-head position D and 156 treated with positions A–C. Before adjudication, the two reviewers agreed on AED position in 196 of 213 included cases (92.0%), indicating substantial inter-reviewer agreement (Cohen’s *κ* = 0.78). The remaining 17 discrepant classifications were resolved by consensus after joint review. The initial rhythm was shockable in 182 patients; the remaining 31 initially had a non-shockable rhythm that subsequently converted before the first AED-delivered shock. Several baseline covariates showed moderate imbalance between position groups, including arrest location, first-rescuer type, AED source, participating center, calendar period, and the collapse-to-AED-arrival interval (absolute standardized mean differences ≥0.20; Table 2 and Supplementary Table S6).

**Table 2:** Clinical cohort baseline characteristics.

Characteristic	Other positions (*n* = 156)	Superior-head position D (*n* = 57)	Group-comparison *p* value	Absolute SMD
Demographic characteristics				
Age, years	62.1 ± 13.4	59.6 ± 14.0	0.246	0.18
Male sex	113 (72.4)	44 (77.2)	0.485	0.11
Arrest and rhythm characteristics				
Arrest location			0.376†	
Home or residence	77 (49.4)	22 (38.6)		**0.22**
Public place	69 (44.2)	31 (54.4)		**0.20**
Other location	10 (6.4)	4 (7.0)		0.02
Initial shockable rhythm (VF/pVT)	132 (84.6)	50 (87.7)	0.570	0.09
Witnessed arrest	117 (75.0)	46 (80.7)	0.385	0.14
Bystander CPR provided	96 (61.5)	39 (68.4)	0.356	0.14
Rescuer and AED characteristics				
Identity of the first rescuer			0.140	
Lay bystander	84 (53.8)	22 (38.6)		**0.31**
Trained non-EMS first responder	48 (30.8)	24 (42.1)		**0.24**
Single EMS or public-safety responder	24 (15.4)	11 (19.3)		0.10
AED source			0.090	
Public-access AED	54 (34.6)	27 (47.4)		**0.26**
First-responder or EMS AED	102 (65.4)	30 (52.6)		**0.26**
Pre-shock interval				
Collapse-to-AED arrival interval, min (*n* = 208)	5.9 [4.8–7.3]	5.5 [4.6–6.5]	0.096	**0.25**
Center and calendar period				
Participating center			0.194	
Center 1	41 (26.3)	20 (35.1)		0.19
Center 2	29 (18.6)	5 (8.8)		**0.29**
Center 3	23 (14.7)	10 (17.5)		0.08
Center 4	23 (14.7)	4 (7.0)		**0.25**
Center 5	19 (12.2)	11 (19.3)		**0.20**
Center 6	21 (13.5)	7 (12.3)		0.04
Calendar period			0.075	
2018–2020	40 (25.6)	8 (14.0)		**0.29**
2021–2022	46 (29.5)	14 (24.6)		0.11
2023–2025	70 (44.9)	35 (61.4)		**0.34**

Key variables relevant to confounding and interpretation of the observational clinical cohort.

Data are presented as mean ± SD, median [IQR], or *n* (%). The age comparison used the Welch independent-samples *t* test; the collapse-to-AED-arrival interval used the Mann–Whitney U test. Categorical variables used the Pearson chi-square test without continuity correction, except arrest location, which used the Fisher–Freeman–Halton exact test (†).

For multicategory variables, the displayed *p* value is the global group-comparison *p* value, whereas SMDs are category-specific. Absolute SMDs for the skewed interval were calculated on the log-transformed scale. Values ≥0.20 are bolded to identify moderate imbalance.

The collapse-to-AED-arrival interval was missing for 5/213 patients (4 in other positions and 1 in position D). Detailed comorbidities, arrest etiology, witness type, AED mode, additional pre-shock intervals, and complete statistical notes are provided in Supplementary Table S6.

AED, automated external defibrillator; CPR, cardiopulmonary resuscitation; EMS, emergency medical services; IQR, interquartile range; pVT, pulseless ventricular tachycardia; SD, standard deviation; SMD, standardized mean difference; VF, ventricular fibrillation.

### Superior-head position D shortened compression-to-first-shock time in the simulation

**Figure 3:**
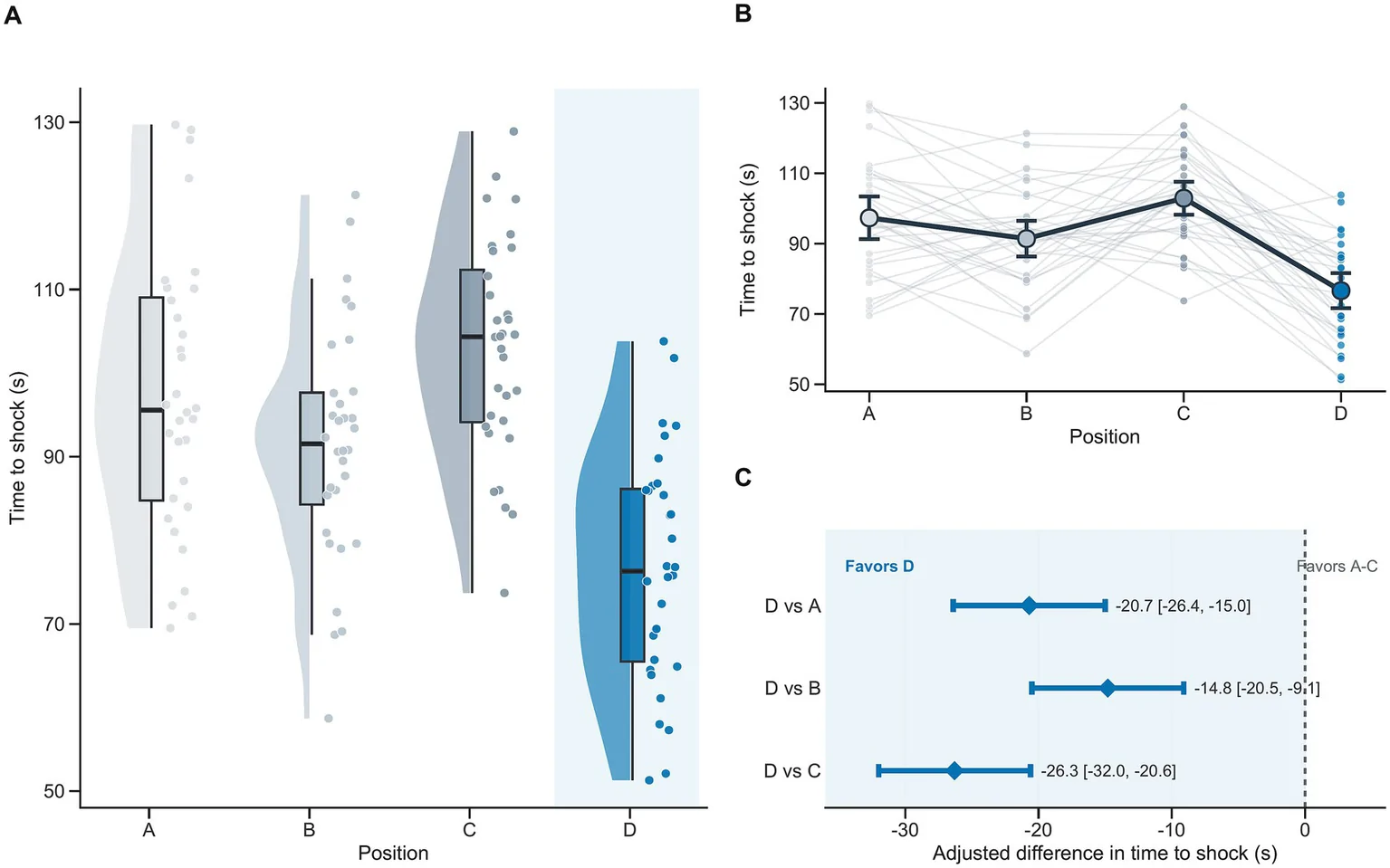
Superior-head position D shortened compression-to-first-shock time in the simulation. Time to shock is the compression-to-first-shock interval (*n* = 32 paired observations per position). **(A)** Raincloud distributions of time to shock by AED position (Friedman *χ*^2^(3) = 38.2, *p* < 0.001). **(B)** Within-participant responses; thin lines connect individual participants and large points show the mean ± 95% CI. **(C)** Adjusted between-position differences from the linear mixed-effects model (position D minus comparator): −20.7 s versus A, −14.8 s versus B, and −26.3 s versus C (all Holm-adjusted *p* < 0.001). Negative values favor position D; data are point estimates with 95% CI. CI, confidence interval.

Compression-to-first-shock time differed across AED positions (Friedman *χ*^2^(3) = 38.2, *p* < 0.001; Figure 3A). Mean times were 97.3 s (SD, 16.8) for position A, 91.4 s (SD, 14.1) for position B, 102.9 s (SD, 13.0) for position C, and 76.6 s (SD, 13.8) for position D (Figures 3A,B, Table 3, and Supplementary Table S2). In the prespecified mixed-effects model, the overall position effect was significant (Wald *χ*^2^(3) = 90.03, *p* < 0.001). Relative to positions A, B, and C, position D shortened compression-to-first-shock time by 20.7 s (95% CI, 15.0–26.4), 14.8 s (95% CI, 9.1–20.5), and 26.3 s (95% CI, 20.6–32.0), respectively; all Holm-adjusted *p* values were <0.001 (Figure 3C, Table 3, and Supplementary Table S2).

**Table 3:** Primary and key secondary simulation outcomes.

Outcome	Position A (*n* = 32)	Position B (*n* = 32)	Position C (*n* = 32)	Position D (*n* = 32)	D vs A adjusted difference (95% CI); *p* value	D vs B adjusted difference (95% CI); *p* value	D vs C adjusted difference (95% CI); *p* value
Compression-to-first-shock time, s	97.3 ± 16.8	91.4 ± 14.1	102.9 ± 13.0	76.6 ± 13.8	−20.7 (−26.4 to −15.0); <0.001	−14.8 (−20.5 to −9.1); <0.001	−26.3 (−32.0 to −20.6); <0.001
AED-related hands-off time, s	17.3 ± 5.4	15.4 ± 3.7	19.1 ± 5.0	11.0 ± 3.0	−6.32 (−8.16 to −4.48); <0.001	−4.44 (−6.28 to −2.60); <0.001	−8.13 (−9.97 to −6.29); <0.001
Chest compression fraction, %	79 ± 4	80 ± 3	78 ± 4	83 ± 3	+3.99 pp. (2.41 to 5.58); <0.001	+2.99 pp. (1.40 to 4.57); <0.001	+5.00 pp. (3.42 to 6.58); <0.001
Raw NASA-TLX workload score	58.2 ± 10.1	55.1 ± 9.5	61.5 ± 11.3	46.5 ± 9.8	−11.70 (−16.30 to −7.11); <0.001	−8.61 (−13.21 to −4.02); <0.001	−15.01 (−19.60 to −10.41); <0.001

Randomized four-sequence crossover AED simulation study (*n* = 32). Values are mean ± SD. Compression-to-first-shock time was defined as the interval from the first chest compression to delivery of the first shock. Adjusted differences are position D minus the comparator and were estimated using linear mixed-effects models including AED position, study period, and randomization sequence as fixed effects and participant as a random intercept. Negative differences favor position D for time-based outcomes and workload; positive differences favor position D for chest compression fraction. CCF contrasts are expressed in percentage points (pp). Within each outcome, the three prespecified position D-versus-comparator *p* values were adjusted using the Holm method; all Holm-adjusted p values remained <0.001. The displayed 95% CIs are nominal, unadjusted Wald confidence intervals.

AED, automated external defibrillator; CCF, chest compression fraction; CI, confidence interval; NASA-TLX, National Aeronautics and Space Administration Task Load Index; SD, standard deviation.

### Position D reduced AED-related interruptions and perceived workload

**Figure 4:**
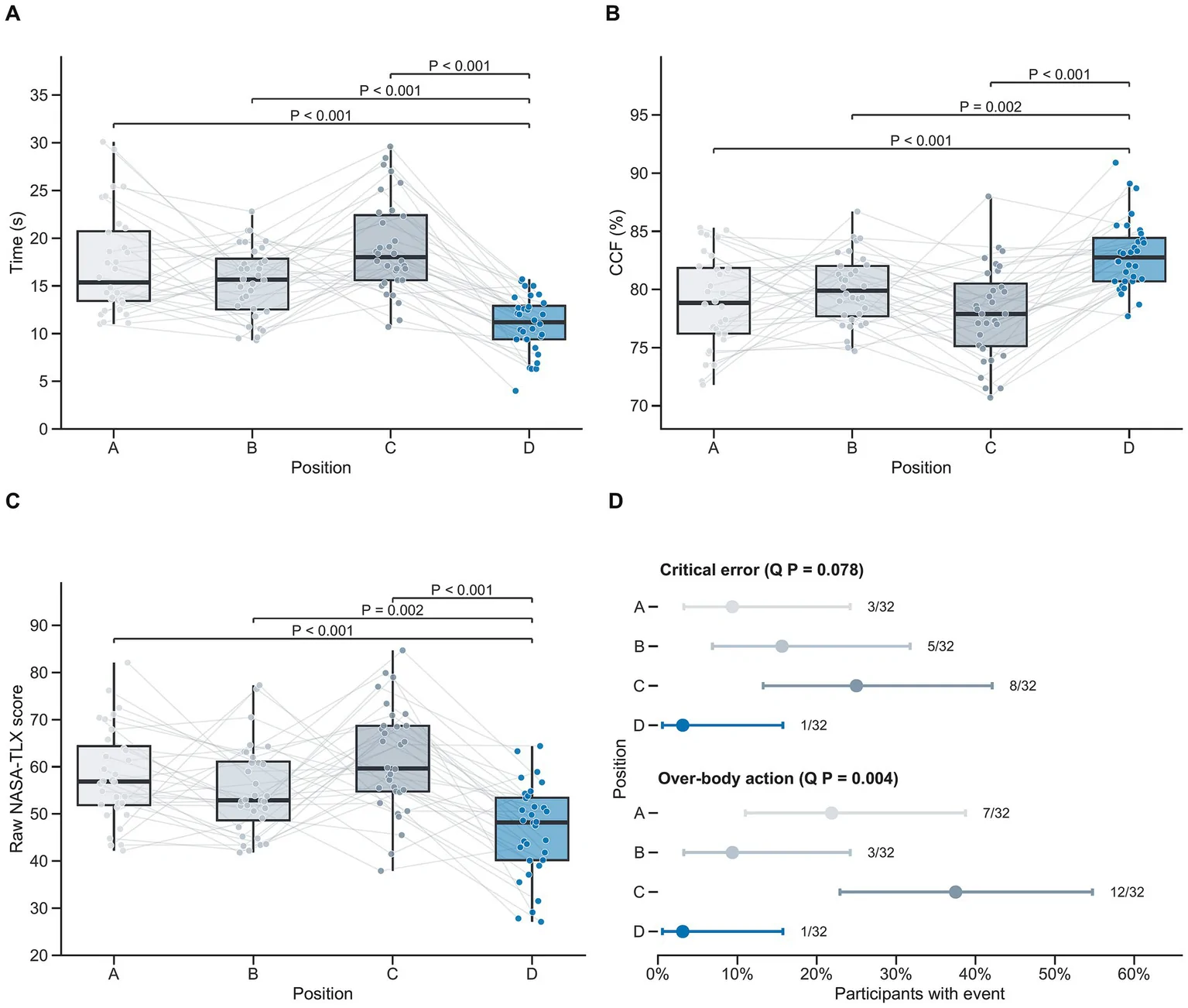
Position D reduced AED-related interruptions and workload without increasing procedural risk. Panels **(A–C)** show simulation outcomes by AED position (*n* = 32 per position); overall comparisons used the Friedman test with Holm-adjusted paired comparisons versus position D. **(A)** AED-related hands-off time. **(B)** Chest compression fraction (CCF). **(C)** Perceived workload (raw NASA-TLX score). **(D)** Procedural-safety events: Incidence of at least one critical error (Cochran *Q p* = 0.078) and of over-body actions (Cochran *Q p* = 0.004); dots show incidence and bars the 95% Wilson CI. The C-versus-D over-body comparison remained significant after Holm adjustment (*p* = 0.010). AED, automated external defibrillator; CCF, chest compression fraction; CI, confidence interval; NASA-TLX, National Aeronautics and Space Administration Task Load Index.

AED-related hands-off time was shortest with position D [11.0 s (SD, 3.0)] compared with positions A [17.3 s (SD, 5.4)], B [15.4 s (SD, 3.7)], and C [19.1 s (SD, 5.0)]. Adjusted position D differences were −6.32 s (95% CI, −8.16 to −4.48) versus A, −4.44 s (95% CI, −6.28 to −2.60) versus B, and −8.13 s (95% CI, −9.97 to −6.29) versus C (all Holm-adjusted *p* < 0.001; Figure 4A, Table 3, and Supplementary Table S3). Chest compression fraction was highest with position D [83% (SD, 3)] compared with A [79% (SD, 4)], B [80% (SD, 3)], and C [78% (SD, 4)]; the corresponding adjusted improvements were 3.99, 2.99, and 5.00 percentage points, respectively (all Holm-adjusted *p* < 0.001; Figure 4B, Table 3, and Supplementary Table S3).

Perceived workload was also lowest with position D. The mean raw National Aeronautics and Space Administration Task Load Index score was 46.5 (SD, 9.8) with position D, compared with 58.2 (SD, 10.1), 55.1 (SD, 9.5), and 61.5 (SD, 11.3) with positions A, B, and C, respectively. Adjusted position D differences were −11.70 points (95% CI, −16.30 to −7.11) versus A, −8.61 points (95% CI, −13.21 to −4.02) versus B, and −15.01 points (95% CI, −19.60 to −10.41) versus C (all Holm-adjusted *p* < 0.001; Figure 4C, Table 3, and Supplementary Table S3). Position effects were observed across all six NASA-TLX domains (all omnibus *p* < 0.001), with partial η*p*^2^ values ranging from 0.220 to 0.371, indicating large effects throughout. The corresponding position D means were 44.8 for mental demand, 51.2 for physical demand, 47.6 for temporal demand, 37.8 for perceived performance, 55.0 for effort, and 42.6 for frustration. Thus, the workload advantage associated with position D was broadly distributed across the six domains rather than being confined to a single component (Supplementary Figure S2).

### Procedural safety and order-related analyses

At least one critical error occurred in 3/32 trials (9.4%) with position A, 5/32 (15.6%) with position B, 8/32 (25.0%) with position C, and 1/32 (3.1%) with position D; the overall paired comparison was not significant (Cochran *Q* = 6.83, *p* = 0.078; Figure 4D and Supplementary Table S4). Over-body action differed across positions (Cochran *Q* = 13.48, *p* = 0.004), occurring in 7/32 trials (21.9%) with position A, 3/32 (9.4%) with position B, 12/32 (37.5%) with position C, and 1/32 (3.1%) with position D. The prespecified C-versus-D comparison remained significant after Holm adjustment (*p* = 0.010), whereas the A-versus-D and B-versus-D comparisons did not (Figure 4D and Supplementary Table S4).

Mean compression-to-first-shock times were 93.7, 92.6, 90.7, and 91.2 s across study periods 1–4, respectively, with no overall period effect (Friedman *χ*^2^(3) = 1.69, *p* = 0.640; Kendall’s *W* = 0.018; Supplementary Figure S1). Mixed-effects analyses similarly showed no clear period or sequence effects for the primary or secondary simulation outcomes, no position-by-period or position-by-sequence interactions, and no linear learning trend (Supplementary Table S5). The period 4-versus-period 3 contrast provided no evidence of a late-period fatigue effect (difference, 0.54 s; 95% CI, −5.19 to 6.26; *p* = 0.854).

### Exploratory clinical associations between position D and process intervals

**Figure 5:**
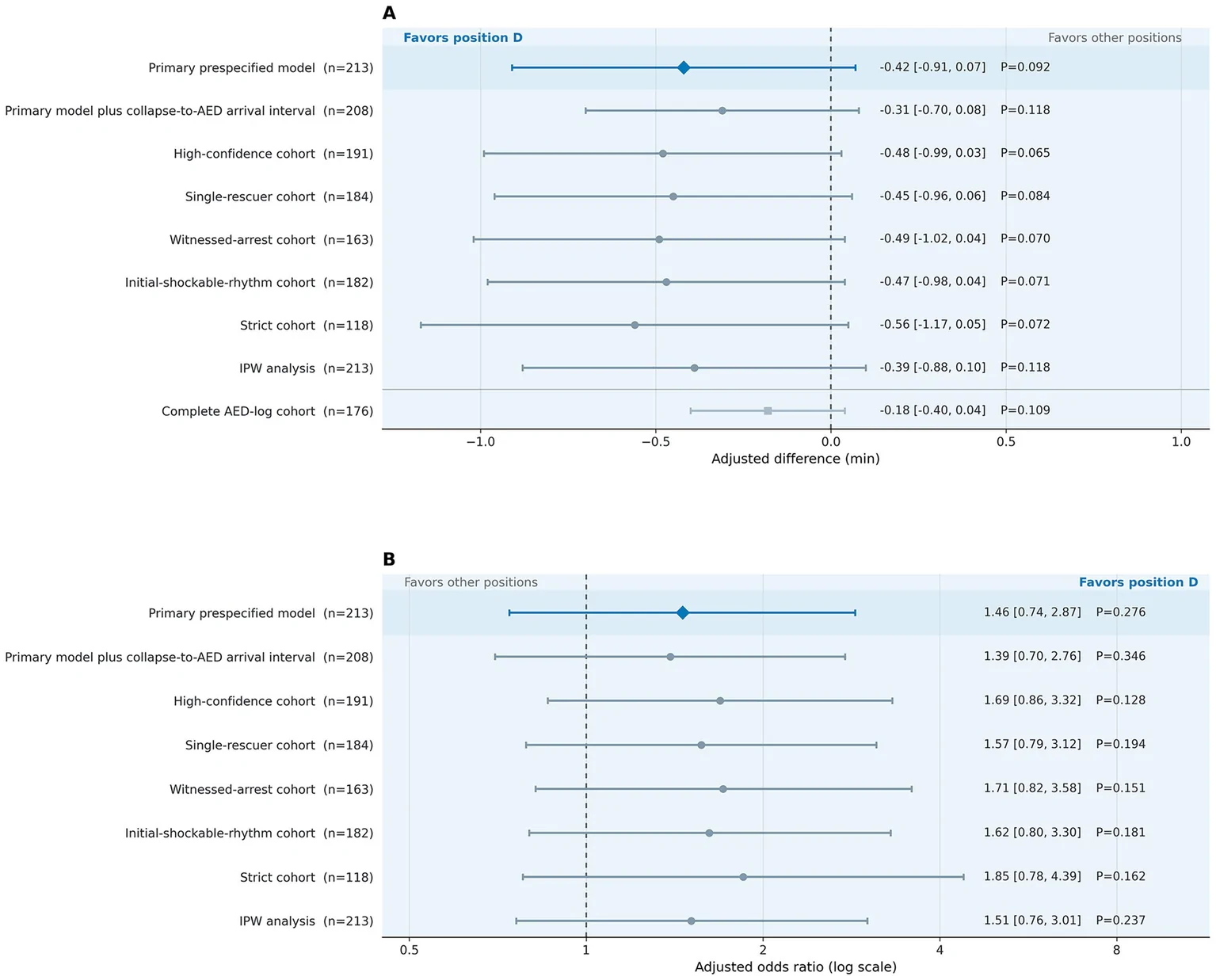
Clinical process intervals consistently favored position D but did not reach statistical significance. Forest plots of adjusted associations between superior-head position D and clinical process intervals (position D versus other positions). **(A)** Adjusted differences in the collapse-to-first-shock interval (minutes) across the primary model and the sensitivity, restricted-cohort, inverse-probability-weighted (IPW), and AED-arrival-to-first-shock analyses; negative values favor position D and all 95% CIs crossed zero. **(B)** Adjusted odds ratios (log scale) for achieving a collapse-to-first-shock interval ≤ 8 min; values > 1 favor position D and all 95% CIs crossed one. Data are point estimates with 95% CI; the analysis-specific *n* is shown for each row. AED, automated external defibrillator; CI, confidence interval; IPW, inverse-probability weighting. Primary and complete AED-log analyses correspond to Table 4 and Supplementary Table S7; additional sensitivity, restricted-cohort, and IPW analyses correspond to Supplementary Table S8.

In the retrospective cohort, the median collapse-to-first-shock interval was 7.7 min (IQR, 7.2–8.6) with position D and 8.2 min (IQR, 7.0–9.8) with other positions. The unadjusted Hodges–Lehmann difference was −0.46 min (95% CI, −0.94 to 0.02; *p* = 0.061). A collapse-to-first-shock interval ≤8 min was achieved in 38/57 patients (66.7%) with position D and 86/156 (55.1%) with other positions (unadjusted OR, 1.63; 95% CI, 0.86–3.07; *p* = 0.132; Table 4). In the primary prespecified model, position D was associated with an adjusted median difference of −0.42 min (95% CI, −0.91 to 0.07; *p* = 0.092) and an adjusted OR of 1.46 (95% CI, 0.74–2.87; *p* = 0.276) for achieving an interval ≤8 min (Figures 5A,B, Table 4, and Supplementary Table S7).

**Table 4:** Clinical process outcomes and adjusted analyses exploratory multicenter clinical cohort: Other AED positions versus superior-head position D.

Outcome or analysis	*n*	Other positions	Position D	Effect estimate (95% CI)	*p* value
Descriptive and unadjusted clinical process outcomes					
Collapse-to-first-shock interval, min	213	8.2 [7.0–9.8]	7.7 [7.2–8.6]	Hodges–Lehmann difference −0.46 min (−0.94 to 0.02)	0.061
Collapse-to-first-shock interval ≤8 min, *n* (%)	213	86 (55.1)	38 (66.7)	Unadjusted OR 1.63 (0.86–3.07)	0.132
AED arrival-to-first-shock interval, min	176	2.25 [1.92–2.72] (*n* = 128)	2.03 [1.78–2.35] (*n* = 48)	Hodges–Lehmann difference −0.20 min (−0.43 to 0.03)	0.088
AED activation-to-pad placement interval, s	176	58 [49–69] (*n* = 128)	51 [43–61] (*n* = 48)	Hodges–Lehmann difference −6.0 s (−12.0 to 0.0)†	0.051
Adjusted analyses					
Primary prespecified model: collapse-to-first-shock interval	213	—	—	Adjusted median difference −0.42 min (−0.91 to 0.07)	0.092
Primary prespecified model: collapse-to-first-shock interval ≤8 min	213	—	—	Adjusted OR 1.46 (0.74–2.87)	0.276
Primary model plus collapse-to-AED arrival interval: collapse-to-first-shock interval	208	—	—	Adjusted median difference −0.31 min (−0.70 to 0.08)	0.118
Primary model plus collapse-to-AED arrival interval: collapse-to-first-shock interval ≤8 min	208	—	—	Adjusted OR 1.39 (0.70–2.76)	0.346
Complete AED-log cohort: adjusted AED arrival-to-first-shock interval	176	—	—	Adjusted median difference −0.18 min (−0.40 to 0.04)	0.109
Witnessed-arrest cohort: collapse-to-first-shock interval	163	—	—	Adjusted median difference −0.49 min (−1.02 to 0.04)	0.070
Witnessed-arrest cohort: collapse-to-first-shock interval ≤8 min	163	—	—	Adjusted OR 1.71 (0.82–3.58)	0.151
Initial-shockable-rhythm cohort: collapse-to-first-shock interval	182	—	—	Adjusted median difference −0.47 min (−0.98 to 0.04)	0.071
Initial-shockable-rhythm cohort: collapse-to-first-shock interval ≤8 min	182	—	—	Adjusted OR 1.62 (0.80–3.30)	0.181

Values are median [IQR] or *n* (%). The collapse-to-first-shock interval was defined as the interval from collapse to delivery of the first shock. The AED arrival-to-first-shock interval was defined as the interval from AED arrival at the patient to delivery of the first shock. The AED activation-to-pad placement interval was defined as the interval from AED activation to completion of pad placement. Unadjusted continuous effects are Hodges–Lehmann location-shift estimates, expressed as position D minus other positions. Adjusted continuous effects are coefficients from median regression at the 50th percentile. For all median-regression models, 95% CIs were obtained using 5,000 patient-level nonparametric bootstrap resamples. Negative time differences favor position D; odds ratios >1 favor position D for achieving a collapse-to-first-shock interval ≤8 min.

The primary prespecified model included age, sex, witnessed arrest, bystander CPR, arrest location, first-rescuer type, participating center, and calendar period. The primary model plus collapse-to-AED arrival interval additionally included the collapse-to-AED arrival interval. The adjusted AED arrival-to-first-shock analysis used the same core covariates in the complete AED-log cohort. Restricted-cohort analyses used the primary adjustment framework.

† The upper confidence limit rounds to 0.0 s, whereas the unrounded two-sided *p* value was 0.051.

AED, automated external defibrillator; CI, confidence interval; IQR, interquartile range; OR, odds ratio.

Among 176 patients with complete AED logs, the median AED-arrival-to-first-shock interval was 2.03 min (IQR, 1.78–2.35; *n* = 48) with position D and 2.25 min (IQR, 1.92–2.72; *n* = 128) with other positions. The adjusted median difference was −0.18 min (95% CI, −0.40 to 0.04; *p* = 0.109; Figure 5A, Table 4, and Supplementary Table S7). The median AED-activation-to-pad-placement interval was 51 s (IQR, 43–61) with position D and 58 s (IQR, 49–69) with other positions, corresponding to an unadjusted Hodges–Lehmann difference of −6.0 s (95% CI, −12.0 to 0.0; *p* = 0.051; Table 4).

### Sensitivity analyses and exploratory patient outcomes

Across sensitivity and restricted-cohort analyses, adjusted differences in the collapse-to-first-shock interval consistently favored position D, ranging from −0.31 to −0.56 min, but every 95% CI crossed zero (Figure 5A and Supplementary Table S8). Adjusted or weighted ORs for achieving an interval ≤8 min ranged from 1.39 to 1.85, with all 95% CIs crossing 1 (Figure 5B and Supplementary Table S8). Leave-one-center-out estimates ranged from −0.36 to −0.48 min and likewise had 95% CIs that crossed zero (Supplementary Figure S3).

Exploratory patient outcomes showed no clear association with AED position. Return of spontaneous circulation occurred in 27/57 patients (47.4%) with position D and 66/156 (42.3%) with other positions (adjusted Firth OR, 1.20; 95% profile penalized-likelihood CI, 0.63–2.27; *p* = 0.578). Adjusted ORs for 24-h survival, 7-day survival, survival to hospital discharge, and favorable neurological outcome at discharge ranged from 1.12 to 1.34; all corresponding 95% CIs included 1 and all *p* values were ≥0.514 (Supplementary Table S9). None of the adjusted clinical analyses reached statistical significance; all confidence intervals for time differences included zero, and all confidence intervals for odds ratios included one.

## Discussion

In this two-part investigation, superior-head AED placement (position D) produced a coherent improvement in the single-rescuer defibrillation workflow under controlled conditions and a directionally similar, although statistically inconclusive, signal in clinical practice. In the randomized crossover simulation, position D shortened compression-to-first-shock time by 14.8–26.3 s relative to the other positions, reduced AED-related hands-off time, increased chest compression fraction, and lowered perceived workload. These benefits were not accompanied by evidence of increased procedural risk: critical errors were numerically least frequent with position D, and over-body actions were uncommon. In the retrospective multicenter cohort, the adjusted point estimate favored position D by 0.42 min, or approximately 25 s, for collapse-to-first-shock time, but the 95% confidence interval included the null. Taken together, the findings identify AED position as a potentially modifiable human-factors component of single-rescuer resuscitation while also defining the need for prospective clinical confirmation.

The simulation findings support a plausible workflow mechanism rather than an isolated reduction in one timing measure. Placing the AED at the superior aspect of the patient’s head may allow the rescuer to reach the controls, pads, and cable from a compact working zone while reducing the need to lean across or move around the patient. The simultaneous reductions in hands-off time and over-body action, together with higher chest compression fraction, are consistent with less task interference during device deployment. Position C may present a different ergonomic constraint during single-rescuer CPR. Because the AED is located near the rescuer’s transition path from the chest-compression position toward the patient’s head, movement to provide rescue breaths may bring the rescuer’s arm or upper body into contact with the AED unit or pad cable, potentially requiring additional cable management or device repositioning. This mechanism may partly explain the higher frequency of over-body actions observed with position C; however, ventilation-related interference was not recorded as a separate outcome and this interpretation therefore remains hypothesis-generating. This matters because the physiological cost of an AED workflow is not limited to the absolute time required to deliver a shock; it also includes the interruption of compressions needed to maintain coronary and cerebral perfusion. Previous clinical studies have associated higher chest compression fraction and shorter preshock or perishock pauses with improved survival after shockable OHCA6, 7. Although the simulation cannot establish an effect on patient outcomes, it shows that a simple spatial modification can improve several process measures that lie on this clinically relevant pathway.

Results extend prior AED human-factors research from device accessibility and interface design to the immediate spatial arrangement of the resuscitation scene. Commercial AEDs differ in analysis, charging, prompting, and hands-off intervals, and device usability can generate substantial variation in time to shock (19). The present findings suggest that operator-device geometry may be an additional source of delay even when the same device and algorithm are used. The workload findings reinforce this interpretation. Simulated CPR imposes substantial cognitive and physical demand, which can rise further when rescuers must perform additional tasks, and simulation research has repeatedly identified human factors and equipment interaction as contributors to delayed defibrillation and unnecessary interruptions (20, 21). Position D may therefore function as a low-complexity ergonomic intervention: it does not change the AED algorithm but may reduce the physical and cognitive coordination required to execute it. All simulation trials in the present study used a JW3201 manikin and a BeneHeart D1 Pro AED platform with the corresponding preconnected adult multifunction defibrillation pads; the manufacturer reports a pad-cable length of approximately 2.1 m for this system. In all trials, the pads were applied in the standard anterolateral configuration, which was completed with the AED at position D under the standardized simulation conditions. Anteroposterior pad placement was not evaluated and may require different cable routing or additional reach, particularly when the patient must be rolled or repositioned. A larger chest circumference or body habitus may similarly increase the distance between the AED unit and the intended pad sites. Holding the equipment constant strengthened the internal comparison by isolating the effect of AED position, but it limits direct extrapolation to other devices. The cable length, pad packaging, control layout, audiovisual interface, and analysis and charging workflow of the BeneHeart D1 Pro may have influenced both the feasibility and magnitude of the observed positional advantage. AEDs with shorter cables or different pad configurations could produce a smaller advantage or make position D impractical. In real-world settings, available floor space, environmental obstacles, airway equipment, noise, contamination, and the number of rescuers may further alter the optimal working arrangement. Accordingly, position D should not be regarded as universally optimal across AED platforms, pad configurations, patient body sizes, or resuscitation environments. Cross-platform simulation incorporating anteroposterior pad placement and a wider range of patient body sizes, followed by prospective real-world validation, is required before broad implementation.

The clinical results require a deliberately non-dichotomous interpretation. The primary adjusted estimate was a median difference of −0.42 min in the collapse-to-first-shock interval with position D (95% CI, −0.91 to 0.07; *p* = 0.092). Thus, the data were compatible with a benefit of approximately 55 s and with an interval approximately 4 s longer, and they did not meet the prespecified threshold for statistical significance. This should not be described as proof of benefit or as a “near-significant” result. Equally, the *p* value does not establish absence of an effect; effect magnitude, precision, design limitations, and consistency across analyses are more informative than a binary significant/non-significant label (22). The appropriate interpretation is therefore an imprecisely estimated but clinically coherent directional signal. Accordingly, the retrospective clinical findings should be considered hypothesis-generating and should not be interpreted as evidence of a confirmed clinical benefit. The adjusted odds ratio of 1.46 for achieving a collapse-to-first-shock interval of 8 min or less pointed in the same direction, but its wide confidence interval (0.74–2.87) likewise permits both benefit and no benefit.

Several observations strengthen the coherence of this clinical signal without resolving its uncertainty. First, the adjusted clinical point estimate of approximately 25 s closely matched the 15–26-s reductions observed in the simulation. This agreement is notable because the two studies used different populations and different interval origins, but it should not be interpreted as equivalence between compression-to-first-shock and collapse-to-first-shock time. Second, intervals closer to AED operation were also directionally shorter: AED-arrival-to-first-shock time was reduced by an adjusted 0.18 min (approximately 11 s), and AED-activation-to-pad-placement time by an unadjusted 6 s. These nested estimates are consistent with at least part of the overall difference arising during device deployment. Third, every restricted-cohort and sensitivity estimate favored position D, as did each leave-one-center-out analysis. Directional stability reduces the likelihood that the estimates were driven by a single center or eligibility definition, but it does not eliminate confounding. Position D was associated with center, calendar period, rescuer type, arrest location, AED source, and the collapse-to-AED-arrival interval. Although multivariable adjustment and inverse-probability weighting addressed measured covariates, they could not account for unmeasured or incompletely recorded factors, including rescuer experience, local training and protocols, AED model and cable configuration, scene geometry, crowding, device-retrieval pathways, and documentation quality. These factors may have influenced both AED placement and time to shock; therefore, the clinical estimates may be biased in either direction and should not be interpreted as causal.

The difference in statistical certainty between the two study components may reflect several factors rather than sample size alone. In the randomized crossover simulation, each participant served as their own control, and the manikin, AED platform, equipment configuration, task sequence, and environment were standardized, thereby reducing between-rescuer and between-scene variability. By contrast, only 57 of the 213 patients in the retrospective cohort were treated with position D, which contributed to the wide confidence interval and limited the precision of the clinical estimate. However, the data do not allow the lack of statistical significance to be attributed solely to the smaller position D group. The primary collapse-to-first-shock interval also incorporated substantial upstream variation that AED position could not influence, including arrest recognition, emergency activation, AED retrieval, rescuer arrival, and bystander or first-rescuer response. Although the models adjusted for witnessed arrest, bystander CPR, arrest location, first-rescuer type, center, and calendar period, more granular differences in recognition delay, rescuer hesitation, CPR quality, AED familiarity, scene geometry, crowding, local protocols, AED model, and cable configuration were not consistently recorded. These factors, together with timestamp uncertainty and heterogeneity across centers and arrest settings, may have increased variability, diluted a position-specific effect, or introduced residual confounding in either direction. A position-dependent difference of approximately 15–25 s may therefore be clinically plausible yet difficult to distinguish from the larger background variability in the collapse-to-first-shock interval.

The AED-arrival-to-first-shock and AED-activation-to-pad-placement intervals were mechanistically more proximal to AED positioning and also directionally favored position D. However, reliable data were available only in the complete AED-log cohort of 176 patients, including 48 treated with position D, and pad-placement timing was not uniformly recorded across devices. These analyses were therefore considered supportive and hypothesis-generating. Exploratory patient outcomes were even less precisely estimated and were influenced by numerous factors occurring after the first shock. Their null-compatible confidence intervals establish neither a clinical benefit nor the absence of an effect from a modest improvement in the pre-shock workflow.

The potential clinical importance of position D lies in its simplicity. Current resuscitation guidance prioritizes early AED use, high-quality compressions, and minimization of avoidable interruptions (2, 3). A standardized superior-head position could be introduced into single-rescuer AED training, dispatcher instructions, equipment-placement protocols, and simulation assessment without purchasing new technology or changing the shock algorithm. Even a modest reduction in a time-critical process may be valuable if it is reproducible, safe, and scalable; however, the historical 8-min defibrillation benchmark should not be interpreted as a target below which further time savings cease to matter (14). Implementation should remain conditional on scene geometry, cable length, airway access, contamination risks, and the number of rescuers present. Position D is most directly supported for the initial single-rescuer phase studied here and should not yet be generalized to established multi-rescuer teams or all clinical environments.

This study has several strengths. The within-participant crossover design controlled for stable differences in technical ability, the counterbalanced sequences limited order effects, and all participants completed all four positions. The absence of clear period, sequence, learning, or fatigue effects supports the internal validity of the positional comparison. The convergence of timing, hands-off, compression-fraction, workload, and movement outcomes provides stronger mechanistic evidence than any single endpoint alone. The multicenter cohort then tested whether the direction and approximate magnitude of the simulation effect were visible in routine care, while prespecified adjustment, restricted cohorts, inverse-probability weighting, and leave-one-center-out analyses examined robustness from complementary angles.

Several limitations should also temper interpretation. The simulation included only 32 predominantly trained healthcare participants and used a manikin under standardized conditions; real arrests involve stress, crowding, variable patient habitus, environmental hazards, and competing clinical tasks. Position D was specifically evaluated for the initial single-rescuer phase, during which no second responder is stationed at the patient’s head and the same rescuer must coordinate chest compressions, basic airway support or ventilation, and AED operation. The superior-head area may therefore remain available for AED placement, provided that airway access is not obstructed. Nevertheless, position D may be impractical in severely confined environments, such as small bathrooms, narrow hallways, or vehicles, and the spatial arrangement may need to be reassessed once additional responders arrive or advanced airway management begins. Accordingly, position D should be regarded as a context-dependent strategy for the initial single-rescuer phase when space and airway access permit, rather than a universal arrangement for every stage or setting of OHCA resuscitation. Participants and assessors could not be blinded to AED position, and the proposed mechanism of reduced reach and cable interference was inferred from process outcomes rather than measured directly. The retrospective cohort was vulnerable to residual confounding because AED position was not randomized and was associated with center, rescuer, scene, AED source, calendar period, and response intervals. Position and timestamp classification depended on the completeness and reliability of existing records, and exclusion of unclassifiable cases may have introduced selection bias. The position D group and the number of patient-outcome events were limited, resulting in wide confidence intervals. Finally, simulation and clinical TTS had different starting points, and the clinical interval included upstream delays that AED position could not influence. Neither component had a formal prospective power calculation. Although the simulation produced precise estimates for the relatively large observed process differences, it may not have detected smaller effects or uncommon safety events. The retrospective cohort, particularly the 57 patients treated with position D, had limited precision for the clinical primary endpoint and patient outcomes. Prospective studies with prespecified effect sizes and sample-size calculations are warranted. The random-number generation procedure, individual allocator, and allocation-concealment process were not fully documented; therefore, the possibility of selection bias cannot be completely excluded. Although AED position was independently classified with substantial pre-adjudication agreement (92.0%; Cohen’s *κ* = 0.78), classification remained dependent on retrospectively available documentation. Residual exposure misclassification cannot be excluded, particularly when direct visual evidence was unavailable, and could have biased the estimated associations in either direction. Excluding 138 otherwise qualifying cases because AED position or first-shock time could not be reliably verified likely reduced exposure and outcome misclassification but may have preferentially selected better-documented resuscitation events. Because comparable baseline data were not consistently available for these excluded records, the direction and magnitude of selection bias could not be quantified, and generalizability may be limited.

Future research should prospectively standardize AED position and timestamp acquisition, preferably using synchronized AED event files and video or dispatch records, and should measure the intermediate steps from AED arrival through activation, pad placement, rhythm analysis, and shock delivery. Dedicated simulation studies should also enrol lay rescuers with varying levels of prior BLS and AED experience, including individuals without previous training. Greater stress and lower familiarity with AED operation, pad placement, and cable management may either increase the value of a simplified spatial arrangement or reduce its benefit when no prior instruction is provided; therefore, the present findings should not be assumed to generalize directly to untrained lay rescuers. Such studies should determine whether brief position D-specific instruction or dispatcher-assisted guidance is required and should assess time to shock, hands-off time, procedural errors, perceived workload, and skill retention. A pragmatic cluster-randomized or stepped implementation study could subsequently test whether training rescuers to place the AED at the superior head reproduces the approximately 20–25-s process advantage suggested here, while monitoring safety, protocol adherence, and patient-centerd outcomes. In conclusion, superior-head AED placement improved the efficiency and workload of simulated single-rescuer CPR. The retrospective clinical estimates were directionally consistent but statistically inconclusive and should be regarded as hypothesis-generating rather than confirmatory. They do not establish clinical benefit, and prospective evaluation is required before position D can be recommended for routine clinical practice.

## Data Availability

The raw data supporting the conclusions of this article will be made available by the authors, without undue reservation.

## Supplementary material

SUPPLEMENTARY FIGURE S1 No period, sequence, or learning effects on time to shock. **(A)** Counterbalanced sequence profiles: mean time to shock (± 95% CI) by study period for each randomisation sequence (*n* = 8 per sequence); letters identify the AED position assigned in each period. **(B)** Overall period effect: mean time to shock by study period across all positions (93.7, 92.6, 90.7, and 91.2 s for periods 1–4; Friedman *χ*²(3) = 1.69, *p* = 0.640; Kendall's W = 0.018). CI, confidence interval.

SUPPLEMENTARY FIGURE S2 Position effects across NASA-TLX workload domains. **(A)** Domain-specific raw NASA-TLX scores (0–100) by AED position (means with 95% CI; *n* = 32 per position); blue labels show Position D means (mental demand 44.8, physical demand 51.2, temporal demand 47.6, performance 37.8, effort 55.0, frustration 42.6). **(B)** Magnitude of position effects expressed as partial *η*² for each domain (all omnibus *p* < 0.001); the dashed line marks the 0.14 large-effect reference. NASA-TLX, National Aeronautics and Space Administration Task Load Index.

SUPPLEMENTARY FIGURE S3 Centre composition and leave-one-centre-out sensitivity analysis. **(A)** Number of patients contributed by each participating centre, stratified by AED position group (Position D versus other positions; total *n* = 213). **(B)** Leave-one-centre-out sensitivity analysis of the adjusted difference in collapse-to-first-shock interval (minutes; Position D minus other positions); estimates remained stable when each centre was omitted in turn, and all 95% CIs crossed zero. CI, confidence interval.
