# Supplementary material for: Superior-head automated external defibrillator placement during single-rescuer cardiopulmonary resuscitation: a randomized crossover simulation study and retrospective multicenter cohort
Source: Front Public Health. 2026 Sep 3;14:1927906. doi: 10.3389/fpubh.2026.1927906 (PMC13581557; doi:10.3389/fpubh.2026.1927906)
Supplement: Supplementary file 1 [file Table_1.DOCX]

**Supplementary Tables S1–S9**

# **Supplementary Table S1. Sequence-specific characteristics of simulation participants**

| **Characteristic** | **Overall (n = 32)** | **Sequence 1 A–B–D–C (n = 8)** | **Sequence 2 B–C–A–D (n = 8)** | **Sequence 3 C–D–B–A (n = 8)** | **Sequence 4 D–A–C–B (n = 8)** | **Descriptive *P* value** |
| --- | --- | --- | --- | --- | --- | --- |
| Age, years | 31.1 ± 3.3 | 28.8 ± 2.9 | 31.6 ± 3.0 | 32.2 ± 3.1 | 31.8 ± 3.4 | 0.125 |
| Female sex | 18 (56.3) | 6 (75.0) | 3 (37.5) | 5 (62.5) | 4 (50.0) | 0.640 |
| Height, cm | 169.0 ± 6.9 | 166.4 ± 7.5 | 170.6 ± 7.0 | 169.2 ± 7.7 | 169.9 ± 5.9 | 0.652 |
| Body mass index, kg/m² | 22.6 ± 1.6 | 21.5 ± 1.6 | 23.4 ± 1.1 | 22.5 ± 1.7 | 23.1 ± 1.5 | 0.086 |
| **Profession** |  |  |  |  |  | 0.538 |
| Physician | 11 (34.4) | 4 (50.0) | 1 (12.5) | 3 (37.5) | 3 (37.5) | — |
| Nurse | 17 (53.1) | 3 (37.5) | 6 (75.0) | 3 (37.5) | 5 (62.5) | — |
| Emergency medical technician | 4 (12.5) | 1 (12.5) | 1 (12.5) | 2 (25.0) | 0 (0.0) | — |
| Clinical experience, years | 7.0 [5.0–9.0] | 4.5 [2.8–6.2] | 7.5 [5.8–9.5] | 8.5 [5.8–10.2] | 7.5 [5.8–9.2] | 0.115 |
| Previous BLS/AED training | 29 (90.6) | 8 (100.0) | 6 (75.0) | 8 (100.0) | 7 (87.5) | 0.587 |
| Time since last BLS/AED training, months* | 10.0 [7.0–16.0] | 9.0 [5.8–13.5] | 11.5 [7.5–18.5] | 11.0 [7.5–16.5] | 11.0 [8.0–14.0] | 0.903 |
| Previous real-life CPR experience | 23 (71.9) | 7 (87.5) | 4 (50.0) | 7 (87.5) | 5 (62.5) | 0.336 |
| Previous real-life AED use | 10 (31.3) | 4 (50.0) | 1 (12.5) | 3 (37.5) | 2 (25.0) | 0.589 |
| Right-hand dominant | 30 (93.8) | 8 (100.0) | 6 (75.0) | 8 (100.0) | 8 (100.0) | 0.226 |

**Notes:** Data are mean ± SD, median [IQR], or n (%). *Time since last training was calculated only among participants with previous BLS/AED training (overall n = 29; Sequence 1, n = 8; Sequence 2, n = 6; Sequence 3, n = 8; Sequence 4, n = 7). Continuous variables were compared using one-way analysis of variance for age, height, and body mass index and the Kruskal–Wallis test for clinical experience and time since last training. Categorical variables were compared using the Fisher–Freeman–Halton exact test. Baseline *P* values are descriptive, were not adjusted for multiplicity, and were not used to assess the success of randomisation or to select covariates.

**Abbreviations:** AED, automated external defibrillator; BLS, basic life support; CPR, cardiopulmonary resuscitation; IQR, interquartile range; SD, standard deviation.

# **Supplementary Table S2. Primary simulation outcome: compression-to-first-shock time**

## **Panel A. Compression-to-first-shock time by AED position**

| **AED position** | **n** | **Mean ± SD, s** | **Median [IQR], s** | **Minimum–maximum, s** |
| --- | --- | --- | --- | --- |
| Position A | 32 | 97.3 ± 16.8 | 95.5 [84.8–109.0] | 69.5–129.7 |
| Position B | 32 | 91.4 ± 14.1 | 91.5 [84.3–97.6] | 58.7–121.3 |
| Position C | 32 | 102.9 ± 13.0 | 104.3 [94.1–112.3] | 73.7–128.9 |
| Position D | 32 | 76.6 ± 13.8 | 76.3 [65.5–86.1] | 51.3–103.8 |

## **Panel B. Supportive repeated-measures analysis of the AED-position effect**

| **Analysis** | **Test statistic** | **Degrees of freedom** | **Greenhouse–Geisser ε** | ***P* value** | **Partial η²** |
| --- | --- | --- | --- | --- | --- |
| Uncorrected repeated-measures ANOVA | F = 28.67 | 3, 93 | — | <0.001 | 0.480 |
| Greenhouse–Geisser-corrected analysis | F = 28.67 | 2.93, 90.81 | 0.976 | <0.001 | 0.480 |

## **Panel C. Prespecified primary linear mixed-effects analysis**

| **Model effect or contrast** | **Adjusted mean difference, s** | **95% CI, s** | **Joint Wald test** | **P value** |
| --- | --- | --- | --- | --- |
| Overall AED-position effect | — | — | χ²(3) = 90.03 | <0.001 |
| Position D vs Position A | −20.7 | −26.4 to −15.0 | — | <0.001 |
| Position D vs Position B | −14.8 | −20.5 to −9.1 | — | <0.001 |
| Position D vs Position C | −26.3 | −32.0 to −20.6 | — | <0.001 |
| Overall study-period effect | — | — | χ²(3) = 1.34 | 0.719 |
| Overall randomisation-sequence effect | — | — | χ²(3) = 1.70 | 0.636 |

**Notes:** Each of the 32 participants completed all four AED-position scenarios, yielding 128 repeated observations. Compression-to-first-shock time was defined as the interval from the first chest compression to delivery of the first shock. The prespecified primary inferential analysis was a linear mixed-effects model including AED position, study period, and randomisation sequence as fixed effects and a participant-specific random intercept; maximum-likelihood estimation was used. Adjusted differences are expressed as Position D minus the comparator, with negative values indicating a shorter compression-to-first-shock time with Position D. Overall position, period, and sequence effects were evaluated using joint Wald χ² tests. The repeated-measures ANOVA was a supportive omnibus analysis; Greenhouse–Geisser correction was applied for potential violation of sphericity. Partial η² represents the proportion of position-plus-error variance attributable to AED position. The three Position D-versus-comparator contrasts were prespecified, and their P values were adjusted using the Holm method across the three contrasts. All Holm-adjusted P values remained <0.001. The displayed 95% CIs are nominal, unadjusted Wald confidence intervals.P values for the three prespecified Position D-versus-comparator contrasts were adjusted using the Holm method. P values for the overall position, period, and sequence effects are unadjusted omnibus P values.

**Abbreviations:** AED, automated external defibrillator; ANOVA, analysis of variance; CI, confidence interval; IQR, interquartile range; SD, standard deviation.

# **Supplementary Table S3. Key secondary simulation outcomes**

## **Panel A. Descriptive results by AED position**

| **Outcome** | **Position A** | **Position B** | **Position C** | **Position D** |
| --- | --- | --- | --- | --- |
| AED-related hands-off time, s | 17.3 ± 5.4 | 15.4 ± 3.7 | 19.1 ± 5.0 | 11.0 ± 3.0 |
| Chest compression fraction, % | 79 ± 4 | 80 ± 3 | 78 ± 4 | 83 ± 3 |
| Raw NASA-TLX workload score | 58.2 ± 10.1 | 55.1 ± 9.5 | 61.5 ± 11.3 | 46.5 ± 9.8 |

## **Panel B. Supportive repeated-measures tests**

| **Outcome** | **F** | **df** | **Uncorrected *P*** | **Greenhouse–Geisser ε** | **Corrected *P*** | **Partial η²** |
| --- | --- | --- | --- | --- | --- | --- |
| AED-related hands-off time, s | 27.84 | 3, 93 | <0.001 | 0.911 | <0.001 | 0.473 |
| Chest compression fraction, % | 14.14 | 3, 93 | <0.001 | 0.931 | <0.001 | 0.313 |
| Raw NASA-TLX workload score | 15.21 | 3, 93 | <0.001 | 0.898 | <0.001 | 0.329 |

## **Panel C. Prespecified Position D contrasts from crossover mixed-effects models**

| **Outcome** | **Contrast** | **Adjusted mean difference** | **95% CI** | **Holm-adjusted P value** |
| --- | --- | --- | --- | --- |
| AED-related hands-off time, s | D vs A | −6.32 s | −8.16 to −4.48 | <0.001 |
| AED-related hands-off time, s | D vs B | −4.44 s | −6.28 to −2.60 | <0.001 |
| AED-related hands-off time, s | D vs C | −8.13 s | −9.97 to −6.29 | <0.001 |
| Chest compression fraction | D vs A | +3.99 percentage points | 2.41 to 5.58 | <0.001 |
| Chest compression fraction | D vs B | +2.99 percentage points | 1.40 to 4.57 | <0.001 |
| Chest compression fraction | D vs C | +5.00 percentage points | 3.42 to 6.58 | <0.001 |
| Raw NASA-TLX workload score | D vs A | −11.70 points | −16.30 to −7.11 | <0.001 |
| Raw NASA-TLX workload score | D vs B | −8.61 points | −13.21 to −4.02 | <0.001 |
| Raw NASA-TLX workload score | D vs C | −15.01 points | −19.60 to −10.41 | <0.001 |

## **Panel D. Overall fixed-effect tests from mixed-effects models**

| **Outcome** | **Position effect, *P* value** | **Period effect, *P* value** | **Sequence effect, *P* value** |
| --- | --- | --- | --- |
| AED-related hands-off time | <0.001 | 0.501 | 0.562 |
| Chest compression fraction | <0.001 | 0.270 | 0.506 |
| Raw NASA-TLX workload score | <0.001 | 0.499 | 0.708 |

**Notes:** Data in Panel A are mean ± SD. Each participant completed all four scenarios. AED-related hands-off time was the component of total hands-off time attributable to AED activation, pad handling and placement, cable management, rhythm analysis, clearing, and shock delivery during the compression-to-first-shock observation window. Chest compression fraction was calculated as chest-compression time divided by the compression-to-first-shock interval. Raw NASA-TLX was the unweighted mean of the six domain scores and ranged from 0 to 100, with higher scores indicating greater perceived workload. Primary inference for the secondary outcomes was based on mixed-effects models including AED position, study period, and randomisation sequence as fixed effects and participant as a random intercept. Repeated-measures ANOVA was supportive. Contrasts are expressed as Position D minus the comparator. Negative values favour Position D for hands-off time and workload; positive values favour Position D for chest compression fraction. Within each outcome, the three prespecified Position D-versus-comparator P values were adjusted using the Holm method. All Holm-adjusted P values remained <0.001. The displayed 95% CIs are nominal, unadjusted Wald confidence intervals.

**Abbreviations:** AED, automated external defibrillator; ANOVA, analysis of variance; CI, confidence interval; NASA-TLX, National Aeronautics and Space Administration Task Load Index; SD, standard deviation.

# **Supplementary Table S4. Safety and procedural-error outcomes**

## **Panel A. Event frequencies and overall paired comparisons**

| **Outcome** | **Position A, n (%)** | **Position B, n (%)** | **Position C, n (%)** | **Position D, n (%)** | **Cochran’s Q** | **Overall paired *P* value** |
| --- | --- | --- | --- | --- | --- | --- |
| At least one critical error | 3 (9.4) | 5 (15.6) | 8 (25.0) | 1 (3.1) | 6.83 | 0.078 |
| Over-body action | 7 (21.9) | 3 (9.4) | 12 (37.5) | 1 (3.1) | 13.48 | 0.004 |

## **Panel B. Prespecified exact paired comparisons with Position D**

| **Outcome** | **Comparison** | **Discordant pairs, comparator-only/D-only** | **Matched OR, comparator vs D (95% exact CI)** | **Exact McNemar *P*** | **Holm-adjusted *P*** |
| --- | --- | --- | --- | --- | --- |
| Critical error | A vs D | 3/1 | 3.00 (0.24–157.49) | 0.625 | 0.625 |
| Critical error | B vs D | 5/1 | 5.00 (0.56–236.49) | 0.219 | 0.438 |
| Critical error | C vs D | 8/1 | 8.00 (1.07–354.98) | 0.039 | 0.117 |
| Over-body action | A vs D | 7/1 | 7.00 (0.90–315.48) | 0.070 | 0.141 |
| Over-body action | B vs D | 3/1 | 3.00 (0.24–157.49) | 0.625 | 0.625 |
| Over-body action | C vs D | 12/1 | 12.00 (1.78–512.97) | 0.003 | 0.010 |

**Notes:** Each participant contributed one paired binary observation under each AED position (n = 32 per position; 128 trials). Operational definitions of critical error and over-body action followed the prespecified simulation and video-adjudication protocol. Overall differences across the four paired positions were assessed using Cochran’s Q test. The three prespecified comparator-versus-D contrasts within each endpoint were evaluated using two-sided exact McNemar tests. Discordant pairs are reported as comparator-only/D-only. Matched ORs are based exclusively on discordant pairs and express the odds of an event with the comparator relative to Position D. Holm adjustment was applied separately across the three comparisons within each endpoint. Exact 95% CIs are unadjusted; therefore, an unadjusted CI may exclude 1 even when the corresponding Holm-adjusted P value exceeds 0.05. Because events were sparse, estimates are imprecise and no multivariable binary-outcome model was fitted.

**Abbreviations:** AED, automated external defibrillator; CI, confidence interval; OR, odds ratio.

# **Supplementary Table S5. Period, sequence, learning, and order-related analyses**

## **Panel A. Global fixed-effect and interaction tests**

| **Outcome** | **Position effect** | **Period effect** | **Sequence effect** | **Position × period interaction** | **Position × sequence interaction** |
| --- | --- | --- | --- | --- | --- |
| Compression-to-first-shock time, s | χ²(3) = 90.03; *P* < 0.001 | χ²(3) = 1.34; *P* = 0.719 | χ²(3) = 1.70; *P* = 0.636 | χ²(9) = 4.18; *P* = 0.899 | χ²(9) = 3.86; *P* = 0.921 |
| AED-related hands-off time, s | χ²(3) = 82.95; *P* < 0.001 | χ²(3) = 2.36; *P* = 0.501 | χ²(3) = 2.05; *P* = 0.562 | χ²(9) = 3.02; *P* = 0.963 | χ²(9) = 3.20; *P* = 0.956 |
| Chest compression fraction | χ²(3) = 42.83; *P* < 0.001 | χ²(3) = 3.92; *P* = 0.270 | χ²(3) = 2.33; *P* = 0.506 | χ²(9) = 3.27; *P* = 0.953 | χ²(9) = 4.63; *P* = 0.865 |
| Raw NASA-TLX workload score | χ²(3) = 45.31; *P* < 0.001 | χ²(3) = 2.37; *P* = 0.499 | χ²(3) = 1.39; *P* = 0.708 | χ²(9) = 5.16; *P* = 0.820 | χ²(9) = 6.08; *P* = 0.731 |

## **Panel B. Adjusted linear trends across successive study periods**

| **Outcome** | **Coefficient per successive period** | **95% CI** | ***P* value** | **Interpretation** |
| --- | --- | --- | --- | --- |
| Compression-to-first-shock time | −0.95 s | −2.76 to 0.86 | 0.305 | No clear linear learning trend detected |
| AED-related hands-off time | −0.05 s | −0.63 to 0.53 | 0.868 | No clear linear learning trend detected |
| Chest compression fraction | −0.21 percentage points | −0.71 to 0.29 | 0.416 | No clear linear learning trend detected |
| Raw NASA-TLX workload score | −0.28 points | −1.74 to 1.17 | 0.701 | No clear linear learning trend detected |

## **Panel C. Additional exploratory order and fatigue checks for compression-to-first-shock time**

| **Analysis** | **Estimate or test** | **95% CI** | ***P* value** | **Interpretation** |
| --- | --- | --- | --- | --- |
| Period 4 vs Period 3 | 0.54 s | −5.19 to 6.26 | 0.854 | No clear late-period fatigue effect detected |
| Position D advantage across sequences | F(3, 28) = 0.23 | — | 0.876 | No clear between-sequence heterogeneity detected |

**Notes:** All analyses included 32 participants and 128 repeated observations. Additive mixed-effects models included AED position, study period, and randomisation sequence as fixed effects and participant as a random intercept. The primary compression-to-first-shock model used maximum-likelihood estimation; secondary-outcome models used restricted maximum-likelihood estimation. Global tests are Wald χ² tests. Position × period models included position, period, their interaction, and participant random intercept but omitted sequence. Position × sequence models included position, sequence, their interaction, and participant random intercept but omitted period. This specification avoided rank deficiency in the balanced four-sequence design. Accordingly, interaction findings are exploratory assessments of order-related heterogeneity and must not be interpreted as independently identified carryover effects. Linear-trend models treated period as continuous and retained position, sequence, and participant random intercept. The Position D advantage analysis assessed whether the within-participant Position D contrast varied across the four randomisation sequences. No formal first-order carryover coefficient was estimated; collectively, the four sequences contained each of the 12 possible ordered first-order transitions once.

**Abbreviations:** AED, automated external defibrillator; CCF, chest compression fraction; CI, confidence interval; NASA-TLX, National Aeronautics and Space Administration Task Load Index.

# **Supplementary Table S6. Baseline characteristics of the clinical cohort**

| **Characteristic** | **Other positions (n = 156)** | **Superior-head Position D (n = 57)** | **Missing, n** | **Descriptive P value** | **Absolute SMD** |
| --- | --- | --- | --- | --- | --- |
| **Patient characteristics** |  |  |  |  |  |
| Age, years | 62.1 ± 13.4 | 59.6 ± 14.0 | 0 | 0.246 | 0.18 |
| Male sex | 113 (72.4) | 44 (77.2) | 0 | 0.485 | 0.11 |
| Hypertension | 69 (44.2) | 22 (38.6) | 0 | 0.462 | 0.11 |
| Diabetes mellitus | 30 (19.2) | 9 (15.8) | 0 | 0.565 | 0.09 |
| Known coronary artery disease | 43 (27.6) | 18 (31.6) | 0 | 0.566 | 0.09 |
| Chronic heart failure | 16 (10.3) | 5 (8.8) | 0 | 0.748 | 0.05 |
| **Presumed arrest aetiology** |  |  | 0 | 0.756 | — |
| Presumed cardiac | 116 (74.4) | 45 (78.9) | — | — | 0.11 |
| Respiratory | 22 (14.1) | 6 (10.5) | — | — | 0.11 |
| Other or unknown | 18 (11.5) | 6 (10.5) | — | — | 0.03 |
| **Arrest and rhythm characteristics** |  |  |  |  |  |
| **Arrest location** |  |  | 0 | 0.376† | — |
| Home or residence | 77 (49.4) | 22 (38.6) | — | — | **0.22** |
| Public place | 69 (44.2) | 31 (54.4) | — | — | **0.20** |
| Other location | 10 (6.4) | 4 (7.0) | — | — | 0.02 |
| Initial shockable rhythm (VF/pVT) | 132 (84.6) | 50 (87.7) | 0 | 0.570 | 0.09 |
| Witnessed arrest | 117 (75.0) | 46 (80.7) | 0 | 0.385 | 0.14 |
| **Witness type** |  |  | 0 | 0.293 | — |
| Bystander-witnessed | 99 (63.5) | 35 (61.4) | — | — | 0.04 |
| First-responder- or EMS-witnessed | 18 (11.5) | 11 (19.3) | — | — | **0.22** |
| Unwitnessed | 39 (25.0) | 11 (19.3) | — | — | 0.14 |
| Bystander CPR provided | 96 (61.5) | 39 (68.4) | 0 | 0.356 | 0.14 |
| **Resuscitation and AED characteristics** |  |  |  |  |  |
| **Identity of first rescuer** |  |  | 0 | 0.140 | — |
| Lay bystander | 84 (53.8) | 22 (38.6) | — | — | **0.31** |
| Trained non-EMS first responder | 48 (30.8) | 24 (42.1) | — | — | **0.24** |
| Single EMS or public-safety responder | 24 (15.4) | 11 (19.3) | — | — | 0.10 |
| **AED source** |  |  | 0 | 0.090 | — |
| Public-access AED | 54 (34.6) | 27 (47.4) | — | — | **0.26** |
| First-responder or EMS AED | 102 (65.4) | 30 (52.6) | — | — | **0.26** |
| Semi-automated AED mode | 139 (89.1) | 52 (91.2) | 0 | 0.652 | 0.07 |
| **Pre-shock time intervals** |  |  |  |  |  |
| Collapse-to-CPR interval, min | 2.4 [1.8–3.5] | 2.2 [1.7–3.0] | 7 | 0.255 | 0.18 |
| Collapse-to-AED arrival interval, min | 5.9 [4.8–7.3] | 5.5 [4.6–6.5] | 5 | 0.096 | **0.25** |
| EMS response interval, min | 8.7 [6.9–10.9] | 8.3 [6.7–9.9] | 4 | 0.260 | 0.18 |
| **Centre and calendar period** |  |  |  |  |  |
| **Participating centre** |  |  | 0 | 0.194 | — |
| Centre 1 | 41 (26.3) | 20 (35.1) | — | — | 0.19 |
| Centre 2 | 29 (18.6) | 5 (8.8) | — | — | **0.29** |
| Centre 3 | 23 (14.7) | 10 (17.5) | — | — | 0.08 |
| Centre 4 | 23 (14.7) | 4 (7.0) | — | — | **0.25** |
| Centre 5 | 19 (12.2) | 11 (19.3) | — | — | **0.20** |
| Centre 6 | 21 (13.5) | 7 (12.3) | — | — | 0.04 |
| **Calendar period** |  |  | 0 | 0.075 | — |
| 2018–2020 | 40 (25.6) | 8 (14.0) | — | — | **0.29** |
| 2021–2022 | 46 (29.5) | 14 (24.6) | — | — | 0.11 |
| 2023–2025 | 70 (44.9) | 35 (61.4) | — | — | **0.34** |

**Notes:** Data are mean ± SD, median [IQR], or n (%). Percentages use the non-missing group denominator. The age comparison used Welch’s independent-samples *t* test; skewed intervals were compared using the Mann–Whitney U test. Categorical variables were compared using Pearson’s χ² test without continuity correction when all expected cell counts were at least 5. A Fisher–Freeman–Halton exact test was used for arrest location (†). *P* values are descriptive and were not adjusted for multiplicity. For multicategory variables, the displayed *P* value is the global group-comparison *P* value, whereas SMDs are category-specific. Absolute SMDs for skewed intervals were calculated on the log-transformed scale; values ≥0.20 are shown in bold. Missing values were collapse-to-CPR interval, 7/213 (3.3%; 5 in other positions and 2 in Position D); collapse-to-AED arrival interval, 5/213 (2.3%; 4 and 1, respectively); and EMS response interval, 4/213 (1.9%; 3 and 1, respectively). All other variables were complete. Overall, 182/213 patients had an initial shockable rhythm; the remaining 31 had an initially non-shockable rhythm with subsequent conversion to a shockable rhythm before the first AED-delivered shock.

**Abbreviations:** AED, automated external defibrillator; CPR, cardiopulmonary resuscitation; EMS, emergency medical services; IQR, interquartile range; pVT, pulseless ventricular tachycardia; SD, standard deviation; SMD, standardised mean difference; VF, ventricular fibrillation.

# **Supplementary Table S7. Sequentially adjusted associations of superior-head Position D with clinical process outcomes**

## **Panel A. Collapse-to-first-shock interval: unadjusted location shift and adjusted conditional median differences**

| **Analysis** | **n** | **Estimand and method** | **Position D estimate, min** | **95% CI, min** | ***P* value** | **Adjustment variables** |
| --- | --- | --- | --- | --- | --- | --- |
| Unadjusted | 213 | Hodges–Lehmann location shift | −0.46 | −0.94 to 0.02 | 0.061 | Position only |
| Age- and sex-adjusted | 213 | Conditional median difference; median regression | −0.45 | −0.93 to 0.03 | 0.066 | Age and sex |
| Event/rescuer-adjusted | 213 | Conditional median difference; median regression | −0.43 | −0.92 to 0.06 | 0.087 | Age, sex, witnessed arrest, bystander CPR, arrest location, and first-rescuer type |
| Primary prespecified model | 213 | Conditional median difference; median regression | −0.42 | −0.91 to 0.07 | 0.092 | Event/rescuer model plus centre and calendar period |
| Primary model plus collapse-to-AED arrival | 208 | Conditional median difference; median regression | −0.31 | −0.70 to 0.08 | 0.118 | Primary model plus collapse-to-AED arrival interval |

## **Panel B. Achievement of a collapse-to-first-shock interval ≤8 min**

| **Analysis** | **n** | **Position D OR** | **95% CI** | ***P* value** | **Adjustment variables** |
| --- | --- | --- | --- | --- | --- |
| Unadjusted | 213 | 1.63 | 0.86–3.07 | 0.132 | Position only |
| Age- and sex-adjusted | 213 | 1.58 | 0.84–2.97 | 0.157 | Age and sex |
| Event/rescuer-adjusted | 213 | 1.49 | 0.77–2.88 | 0.235 | Age, sex, witnessed arrest, bystander CPR, arrest location, and first-rescuer type |
| Primary prespecified model | 213 | 1.46 | 0.74–2.87 | 0.276 | Event/rescuer model plus centre and calendar period |
| Primary model plus collapse-to-AED arrival | 208 | 1.39 | 0.70–2.76 | 0.346 | Primary model plus collapse-to-AED arrival interval |

## **Panel C. Position-proximal clinical process outcome**

| **Outcome** | **Analysis population** | **n** | **Effect measure** | **Position D estimate** | **95% CI** | ***P* value** | **Adjustment variables** |
| --- | --- | --- | --- | --- | --- | --- | --- |
| AED arrival-to-first-shock interval | Complete AED-log cohort | 176 | Adjusted conditional median difference, min | −0.18 | −0.40 to 0.04 | 0.109 | Age, sex, witnessed arrest, bystander CPR, arrest location, first-rescuer type, centre, and calendar period |

**Notes:** Estimates are expressed as Position D minus other positions. Negative continuous estimates indicate a shorter interval with Position D. The unadjusted continuous estimate is a Hodges–Lehmann location-shift estimate; adjusted continuous estimates are conditional median differences from median-regression models. Because these are related but non-identical estimands, changes across the rows of Panel A should not be interpreted solely as attenuation caused by covariate adjustment. For all median-regression models, 95% CIs were obtained using 5,000 nonparametric bootstrap resamples. Binary outcomes were analysed using logistic regression; ORs >1 favour Position D for achieving a collapse-to-first-shock interval ≤8 min. Centre and calendar period were included as fixed categorical terms; no centre-level random effect was fitted. The primary model did not include initial rhythm; its influence was evaluated in the prespecified initial-shockable-rhythm restricted analysis in Supplementary Table S8. The complete AED-log cohort comprised 128 patients in other positions and 48 in Position D. The collapse-to-first-shock interval begins at collapse and reflects the overall resuscitation process, whereas the AED arrival-to-first-shock interval is position-proximal. Neither clinical interval is interchangeable with the simulation compression-to-first-shock time.

**Abbreviations:** AED, automated external defibrillator; CI, confidence interval; CPR, cardiopulmonary resuscitation; OR, odds ratio.

# **Supplementary Table S8. Sensitivity and restricted-cohort analyses of clinical process outcomes**

## **Panel A. Continuous clinical process intervals**

| **Analysis** | **Outcome** | **n (Other/D)** | **Effect measure** | **Position D estimate, min** | **95% CI, min** | ***P* value** |
| --- | --- | --- | --- | --- | --- | --- |
| Primary prespecified model | Collapse-to-first-shock interval | 213 (156/57) | Adjusted conditional median difference | −0.42 | −0.91 to 0.07 | 0.092 |
| Primary model plus collapse-to-AED arrival | Collapse-to-first-shock interval | 208 (152/56) | Adjusted conditional median difference | −0.31 | −0.70 to 0.08 | 0.118 |
| High-confidence position cohort | Collapse-to-first-shock interval | 191 (140/51) | Adjusted conditional median difference | −0.48 | −0.99 to 0.03 | 0.065 |
| Single-rescuer-before-shock cohort | Collapse-to-first-shock interval | 184 (134/50) | Adjusted conditional median difference | −0.45 | −0.96 to 0.06 | 0.084 |
| Witnessed-arrest cohort | Collapse-to-first-shock interval | 163 (117/46) | Adjusted conditional median difference | −0.49 | −1.02 to 0.04 | 0.070 |
| Initial-shockable-rhythm cohort | Collapse-to-first-shock interval | 182 (132/50) | Adjusted conditional median difference | −0.47 | −0.98 to 0.04 | 0.071 |
| Strict position-specific cohort | Collapse-to-first-shock interval | 118 (85/33) | Adjusted conditional median difference | −0.56 | −1.17 to 0.05 | 0.072 |
| Inverse-probability-weighted analysis | Collapse-to-first-shock interval | 213 (156/57) | IPW-weighted mean difference | −0.39 | −0.88 to 0.10 | 0.118 |
| Complete AED-log cohort | AED arrival-to-first-shock interval | 176 (128/48) | Adjusted conditional median difference | −0.18 | −0.40 to 0.04 | 0.109 |

## **Panel B. Achievement of a collapse-to-first-shock interval ≤8 min**

| **Analysis** | **n (Other/D)** | **Adjusted or weighted OR** | **95% CI** | ***P* value** |
| --- | --- | --- | --- | --- |
| Primary prespecified model | 213 (156/57) | 1.46 | 0.74–2.87 | 0.276 |
| Primary model plus collapse-to-AED arrival | 208 (152/56) | 1.39 | 0.70–2.76 | 0.346 |
| High-confidence position cohort | 191 (140/51) | 1.69 | 0.86–3.32 | 0.128 |
| Single-rescuer-before-shock cohort | 184 (134/50) | 1.57 | 0.79–3.12 | 0.194 |
| Witnessed-arrest cohort | 163 (117/46) | 1.71 | 0.82–3.58 | 0.151 |
| Initial-shockable-rhythm cohort | 182 (132/50) | 1.62 | 0.80–3.30 | 0.181 |
| Strict position-specific cohort | 118 (85/33) | 1.85 | 0.78–4.39 | 0.162 |
| Inverse-probability-weighted analysis | 213 (156/57) | 1.51 | 0.76–3.01 | 0.237 |

**Notes:** Continuous effects are expressed as Position D minus other positions; negative estimates favour Position D. ORs >1 favour Position D for achieving a collapse-to-first-shock interval ≤8 min. The primary model included age, sex, witnessed arrest, bystander CPR, arrest location, first-rescuer type, centre, and calendar period. The collapse-to-AED-arrival-adjusted model additionally included the collapse-to-AED arrival interval. For all median-regression models, 95% CIs were obtained using 5,000 nonparametric bootstrap resamples. Restricted-cohort models followed the primary adjustment framework but excluded covariates with no within-cohort variation. The high-confidence cohort excluded 22 records with low-confidence position classification. The single-rescuer-before-shock cohort excluded 29 patients in whom a second rescuer arrived before the first shock. The initial-shockable-rhythm cohort was prespecified because the full cohort included patients with an initially non-shockable rhythm who subsequently converted to a shockable rhythm. The strict cohort was the intersection of witnessed arrest, initial shockable rhythm, high-confidence position classification, and no second rescuer before the first shock. The IPW continuous estimand was the weighted mean difference in collapse-to-first-shock interval between Position D and other positions. Stabilised weights were truncated at the 1st and 99th percentiles. The complete AED-log analysis used the primary clinical adjustment set. All sensitivity and restricted-cohort analyses were intended to assess robustness rather than provide independent confirmatory evidence.

**Abbreviations:** AED, automated external defibrillator; CI, confidence interval; CPR, cardiopulmonary resuscitation; IPW, inverse-probability weighting; OR, odds ratio.

# **Supplementary Table S9. Exploratory patient outcomes**

| **Outcome** | **Other positions (n = 156), n/N (%)** | **Superior-head Position D (n = 57), n/N (%)** | **Unadjusted OR (95% CI)** | **Unadjusted P value** | **Adjusted Firth OR (95% profile penalised-likelihood CI)** | **Profile penalised-likelihood P value** |
| --- | --- | --- | --- | --- | --- | --- |
| Return of spontaneous circulation | 66/156 (42.3) | 27/57 (47.4) | 1.23 (0.67–2.26) | 0.510 | 1.20 (0.63–2.27) | 0.578 |
| 24-hour survival | 39/156 (25.0) | 17/57 (29.8) | 1.28 (0.65–2.50) | 0.479 | 1.26 (0.62–2.55) | 0.519 |
| 7-day survival | 20/156 (12.8) | 10/57 (17.5) | 1.45 (0.63–3.31) | 0.382 | 1.34 (0.55–3.27) | 0.514 |
| Survival to hospital discharge | 14/156 (9.0) | 6/57 (10.5) | 1.19 (0.44–3.27) | 0.731 | 1.12 (0.36–3.45) | 0.844 |
| Favourable neurological outcome at hospital discharge (CPC 1–2) | 12/156 (7.7) | 5/57 (8.8) | 1.15 (0.39–3.43) | 0.797 | 1.18 (0.34–4.06) | 0.793 |

**Notes:** Values are n/N (%). Unadjusted ORs, log-Wald 95% CIs, and two-sided P values were calculated from the reported 2 × 2 tables. ORs >1 indicate higher odds of the outcome with Position D. Adjusted estimates were obtained using parsimonious Firth penalised logistic-regression models including age, witnessed-arrest status, and initial shockable rhythm. Adjusted 95% CIs and two-sided P values were derived using profile penalised-likelihood inference. Centre was not included because of sparse events and the prespecified parsimonious modelling strategy. Favourable neurological outcome was defined as CPC 1–2 at hospital discharge. Patient outcomes were exploratory and underpowered; all adjusted and unadjusted CIs included the null value. No multiplicity adjustment was applied. These findings should not be interpreted as evidence of improved survival or neurological outcome.

**Abbreviations:** CI, confidence interval; CPC, Cerebral Performance Category; OR, odds ratio.
